# The adverse events of haematopoietic stem cell transplantation are associated with gene polymorphism within human leukocyte antigen region

**DOI:** 10.1038/s41598-020-79369-w

**Published:** 2021-01-14

**Authors:** Ding-Ping Chen, Ying-Hao Wen, Po-Nan Wang, Ai-Ling Hour, Wei-Tzu Lin, Fang-Ping Hsu, Wei-Ting Wang

**Affiliations:** 1 Department of Laboratory Medicine, Linkou Chang Gung Memorial Hospital, Taoyuan City, 333 Taiwan; 2 Department of Medical Biotechnology and Laboratory Science, College of Medicine, Chang Gung University, Taoyuan County, Taiwan; 3 Graduate Institute of Biomedical Sciences, College of Medicine, Chang Gung University, Taoyuan County, Taiwan; 4 Graduate Institute of Clinical Medical Sciences, College of Medicine, Chang Gung University, Taoyuan, Taiwan; 5 Division of Hematology-Oncology, Department of Internal Medicine, Chang Gung Memorial Hospital, Taoyuan, Taiwan; 6 Department of Life Science, Fu Jen Catholic University, Taipei, Taiwan

**Keywords:** Prognostic markers, Genetics

## Abstract

Adverse reactions may still occur in some patients after receiving haematopoietic stem cell transplantation (HSCT), even when choosing a human leukocyte antigen (HLA)-matched donor. The adverse reactions of transplantation include disease relapse, graft-versus-host disease (GVHD), mortality and CMV infection. However, only the relapse was discussed in our previous study. Therefore, in this study, we investigated the correlation between the gene polymorphisms within the HLA region and the adverse reactions of post-HSCT in patients with acute leukaemia (n = 176), where 72 patients were diagnosed with acute lymphocytic leukaemia (ALL) and 104 were acute myeloid leukaemia (AML). The candidate single nucleotide polymorphisms were divided into three models: donor, recipient, and donor-recipient pairs and the data of ALL and AML were analysed individually. Based on the results, we found 16 SNPs associated with the survival rates, the risk of CMV infection, or the grade of GVHD in either donor, recipient, or donor-recipient matching models. In the ALL group, the rs209132 of TRIM27 in the donor group was related to CMV infection (p = 0.021), the rs213210 of RING1 in the recipient group was associated with serious GVHD (p = 0.003), and the rs2227956 of HSPA1L in the recipient group correlated with CMV infection (p = 0.001). In the AML group, the rs3130048 of BAG6 in the donor-recipient pairs group was associated with serious GVHD (p = 0.048). Moreover, these SNPs were further associated with the duration time of survival after transplantation. These results could be applied to select the best donor in HSCT.

## Introduction

Haematopoietic stem cell transplantation (HSCT) is one of the treatments to cure various kinds of haematologic diseases and cancers^1^, such as acute myeloid leukaemia (AML) and acute lymphocytic leukaemia (ALL). HSCT’s treatment principle is to reconstruct the haematopoietic and immune system^2^ by transplanting the healthy stem cells into patients, and the bone marrow, cord blood and peripheral blood could all be used as the source of stem cells. The human leukocyte antigen (HLA) system (also knowns as major histocompatibility complex, MHC) is located on chromosome 6p21, in which the large genetic diversity is related to graft rejection^3^. Moreover, there are many immune-regulatory genes distributed in this region^4^, which play an important role in heterogeneous HSCT^5,6^. Therefore, HLA-typing and HLA-matching (major on the HLA-A, -B, -C, -DR and -DQ alleles) between the donor and recipient must be performed before receiving allogeneic HSCT. The HLA-matched donor had a generally better prognosis^7^, and the risk of graft-versus-host disease (GVHD) was lower than those with an HLA-mismatched donor^8^. Unfortunately, even selecting an HLA-matched donor, the disease relapse and other poor outcomes might still occur after receiving HSCT.

In 2013, Petersdorf et al. demonstrated that several SNPs in non-classical HLA genes could potentially affect the effectiveness of HSCT^9^. We used these SNPs (rs2244546, rs394657, rs429916, rs915654, rs2075800, rs2242656, rs107822, rs209130 and rs2071479) as sourced SNPs to look for candidate SNPs, which were within the 500 bp flanking genomic regions of these sourced SNPs. In our previous studies, we investigated the correlation between these candidate SNPs and disease relapse of post-HSCT, and the results had been reported in *peerj* (2018)^10^ and *scientific reports* (2019)^11^, respectively. These studies supported that the outcomes of allo-HSCT might be affected by genes within the HLA system besides HLA-A, -B, -C, -DR and -DQ.

However, HSCT’s adverse reactions include mortality, GVHD and cytomegalovirus (CMV) infection, besides disease relapse. In this study, we expanded these two studies mentioned above by increasing the sample of patients with AML or ALL who receiving HSCT and investigating the relationship between these adverse reactions of HSCT mentioned above and gene polymorphisms within the HLA region.

## Results

The clinical characteristics of the patients were summarised in Table 1. The 176 patients were enrolled in this study, including 104 patients with AML and 72 patients with ALL. Additionally, AML patients were older (30.64 ± 11.43 years old) than ALL patients (21.86 ± 14.12 years old), and the male to the female gender ratio of these patients just was 1:1. All patients’ survival rate was 52%, the relapse rate was 74%, CMV infection rate was 56% and the rate of getting severe GVHD was 27%. Furthermore, in this study, the most common stem cell source was peripheral blood (70.5%), and the most common conditioning regimen was chemotherapy (59.1%).

**Table 1:** The clinical characteristics of the patient who received allogeneic HSCT.

Characteristics of patient	Number of patients
**Number of patients**	176
AML	104 (59.1%)
ALL	72 (40.9%)
Male:female	88:88
**Age**	
AML	30.64 ± 11.43 (range from 1 to 66 years old)
ALL	21.86 ± 14.12 (range from 0.8 to 54 years old)
Survival rate	52%
Relapse rate	74%
**GVHD**	
Non-GVHD	19
Grade 1–2	97
Grade 3–4	48
Chronic	12
CMV infection	99
**Source of cells**	
PB	124
BM	52
**Conditioning regimen**	
Only chemotherapy	104
Only radiotherapy	11
Chemoradiotherapy	55
NA	6

Conditioning regimen is defined as emptying bone marrow space for new bone marrow cell growth after the destruction of bone marrow and cancer cells by chemotherapy or radiotherapy before transplantation.

*PB* peripheral blood, *BM* bone marrow.

In Table 2, regarding the correlation between the three adverse reactions of HSCT and the candidate SNPs in ALL group, the rs209132 of *TRIM27* gene was associated with the risk of CMV infection (p = 0.021), and the rs213210 of the *RING1* gene was related to the risk of GVHD grade 3–4 (p = 0.003) in donor group. In the recipient group, 3 SNPs correlated with CMV infection, including of the rs9282369 of the *HLA-DOA* gene (p = 0.014), the rs2227956 of *HSPA1L* gene (p = 0.001) and the rs3130048 of the *BAG6* gene (p = 0.035). Moreover, 2 SNPs were associated with the grade 3–4 of GVHD, including the rs213210 of *RING1* gene (p = 0.024) and rs139791445 of *TRIM27* gene (p = 0.044). For the donor-recipient pairs group, only the rs209130 of *TRIM27* gene was related to the risk of GVHD grade 3–4 (p = 0.036), in which the matched gene polymorphism of rs209130 in donor-recipient pairs had a lower probability for getting grade3-4 GVHD than those who were unmatched (OR = 0.333, 95% CI 0.117–0.946).

**Table 2:** The SNPs significant association with three adverse reactions in ALL group.

SNP	Gene	Chromosome position (bp)	Source	Outcome /status	Recipient Genotype frequency (%)				Test	*p*-value	OR (95% CI)
**Donor group**											
rs209132	TRIM27	Chr 6: 28899705	rs209130	CMV	A/A	A/G		G/G			
	~ 3 k downstream			Yes	2	8		25	D	0.021	0.320 (0.120–0.857)
				No	3	17		16			
rs213210	RING1	Chr 6:33208047	rs107822	GVHD3-4	A/A	A/G		G/G			
	Promoter			Yes	6	5		13	D	0.003	0.193 (0.064–0.588)
				No	12	23		8			
**Recipient group**											
rs9282369	HLA-DOA	Chr 6: 33011011–33011018	rs9276982	CMV	−/−	−/T		T/T			
	Promoter			Yes	8	17		9	R	0.014	6.300 (1.252–31.703)
				NO	12	23		2			
rs2227956	HSPA1L	Chr 6: 31810495	rs2075800	CMV	A/A	A/G		G/G			
	Exon			Yes	28	4		3	D	0.001	0.190 (0.066–0.546)
				No	16	19		2			
rs3130048	BAG6	Chr 6: 31645962	rs2242656	CMV	C/C	C/T		T/T			
	Intron			Yes	5	11		19	D	0.035	0.356 (0.135–0.939)
				No	8	18		11			
rs213210	RING1	Chr 6:33208047	rs107822	GVHD3-4	A/A	A/G		G/G			
	Promoter			Yes	3	10		11	D	0.024	0.287 (0.094–0.873)
				No	8	25		8			
rs139791445	TRIM27	Chr 6: 28900314	rs209130	GVHD3-4	C/C	C/G		G/G			
	~ 3 k downstream			Yes	23	3			D/A	0.044	NA
				No	46						

SNP	Gene	Chromosome position (bp)	Source	Outcome /status	Genotypes between the Donor-Recipient Pair				Test	*p*-value	OR (95% CI)
**Donor-recipient pairs**											
rs209130	TRIM27	Chr 6: 28900023	rs209130	GVHD3-4	Matched		Unmatched				
	~ 3 k downstream			Yes	14		12		Chi-square	0.036	0.333 (0.117–0.946)
				No	35		10				

D: dominant model (AA vs. Aa + aa); R: recessive model (AA + Aa vs. aa); A: additive model (AA vs. Aa vs. aa), in which ‘A’ was defined as a higher frequency allele and the lower was ‘a’. Fisher’s exact test was used when more than 20% of cells had an expected count of less than 5 for the Chi-square test. The unmatched was used as a standard for odds ratio here. In other words, the chance for getting CMV infection, severe GVHD and survival in the matched genotype in donor-recipient pairs compared to those unmatched.

The relation between the three adverse reactions of HSCT and the candidate SNPs in the AML group was demonstrated in Table 3. Two SNPs of *HLA-DOB* had significantly correlated with adverse reactions in the donor group, where one of the SNPs was associated with CMV infection (rs11244, p = 0.019), and the other was related to survival rate (rs17220087, p = 0.047). Additionally, the rs209131 of *TRIM27* was associated with CMV infection (p = 0.034), the rs2518028 of *HCP5* was related to the survival rate (p = 0.026) and the rs1536215 of *TRIM27* was related to GVHD grade 3–4 (p = 0.021). In the recipient group, the rs209132 and rs209131 of *TRIM27* (p = 0.022 and 0.042, respectively) and the rs2070120 and the rs17213693 of *HLA-DOB* (p = 0.026 and 0.043, respectively) were associated with the risk of CMV infection. Moreover, the rs79327197 of *HLA-DOA* was related to survival rate, and it had higher mortality frequency in patients with minor allele, in which the allele with higher frequency in ALL or AML population was defined as a major allele, and the lower was minor allele (minor allele = G-allele; p = 0.008, OR = 0.217, 95% CI 0.065–0.720). In the donor-recipient pairs group, the rs209131 of *TRIM27* (p = 0.020) and the rs17213693 of *HLA-DOB* (p = 0.044) gene polymorphism matched or unmatched were associated with CMV infection. And the patients had lower survival when the gene polymorphism of rs107822 in the *RING1* promoter region was matched in donor-recipient pairs (p = 0.016, OR = 0.368, 95% CI 0.161–0.838). Furthermore, the patients had a lower risk of getting severe GVHD when the genotype of rs3130048 in the intron region of *BAG6* was matched in donor-recipient pairs (p = 0.048, OR = 0.302, 95% CI 0.099–0.920).

**Table 3:** The SNPs significant association with three adverse reactions in AML group.

SNP	Gene	Chromosome position (bp)	Source	Outcome /status	Recipient Genotype frequency (%)				Test	*p*-value	OR (95% CI)
**Donor group**											
rs11244	HLA-DOB	Chr 6: 32812947	rs2071479	CMV	A/A	A/G		G/G			
	3′UTR			Yes		21		41	D	0.019	0.379 (0.167–0.858)
				No	2	21		17			
rs209131	TRIM27	Chr 6: 28899978	rs209130	CMV	A/A	A/G		G/G			
	~ 3 k downstream			Yes	16	28		19	R	0.034	0.322 (0.109–0.946)
				No	10	26		5			
rs2518028	HCP5	Chr6: 31468270	rs2244546	Survival	C/C	C/T		T/T			
	~ 2.5 k downstream			Yes	38	15		1	R	0.026	8.833 (1.045–74.633)
				No	29	13		7			
rs17220087	HLA-DOB	Chr 6: 32813299	rs2071479	Survival	A/A	A/C		C/C			
	3′UTR			Yes		3		50	D/A	0.047	3.750 (0.952–14.775)
				No		9		40			
rs1536215	TRIM27	Chr 6: 28900138	rs209130	GVHD3-4	C/C	C/G		G/G			
	~ 3 k downstream			Yes	9	12		1	D	0.021	3.056 (1.159–8.056)
				No	55	23		3			
**Recipient group**											
rs209132	TRIM27	Chr 6: 28899705	rs209130	CMV	A/A	A/G		G/G			
	~ 3 k downstream			Yes	2	23		37	D	0.022	0.390 (0.173–0.879)
				No	5	21		15			
rs209131	TRIM27	Chr 6: 28899978	rs209130	CMV	A/A	A/G		G/G			
	~ 3 k downstream			Yes	7	34		21	R	0.042	0.347 (0.122–0.989)
				No	11	23		7			
rs2070120	HLA-DOB	Chr 6: 32813137	rs2071479	CMV	A/A	A/G		G/G			
	3′UTR			Yes		11		52	D/A	0.026	0.118 (0.015–0.954)
				No		1		40			
rs17213693	HLA-DOB	Chr 6: 32813344	rs2071479	CMV	C/C	C/G		G/G			
	Intron			Yes	1	11		51	D	0.043	4.471 (0.944–21.163)
				No		2		38			
rs79327197	HLA-DOA	Chr 6: 33010635	rs9276982	Survival	A/A	A/G		G/G			
	Promoter			Yes	51	4			D/A	0.008	0.217 (0.065–0.720)
				NO	36	13					

SNP	Gene	Chromosome position (bp)	Source	Outcome /status	Genotypes between the Donor-Recipient Pairs				Test	*p*-value	OR (95% CI)
**Donor-recipient pairs**											
rs209131	TRIM27	Chr 6: 28899978	rs209130	CMV	Matched		Unmatched				
	~ 3 k downstream			Yes	34		28		Chi-square	0.016	0.342 (0.140–0.834)
				No	32		9				
rs17213693	HLA-DOB	Chr 6: 32813344	rs2071479	CMV	Matched		Unmatched				
	Intron			Yes	50		12		Chi-square	0.044	0.225 (0.048–1.068)
				No	37		2				
rs107822	RING1	Chr 6: 33207798	rs107822	Survival	Matched		Unmatched				
	Promoter			Yes	25		28		Chi-square	0.016	0.368 (0.161–0.838)
				No	34		14				
rs3130048	BAG6	Chr 6: 31645962	rs2242656	GVHD3-4	Matched		Unmatched				
	Intron			Yes	15		7		Fisher’s exact test	0.048	0.302 (0.099–0.920)
				No	71		10				

D: dominant model (AA vs. Aa + aa); R: recessive model (AA + Aa vs. aa); A: additive model (AA vs. Aa vs. aa), in which ‘A’ was defined as a higher frequency allele and the lower was ‘a’. Fisher’s exact test was used when more than 20% of cells had an expected count of less than 5 for the Chi-square test. The unmatched was used as a standard for odds ratio here. In other words, the chance for getting CMV infection, severe GVHD and survival in the matched genotype in donor-recipient pairs compared to those unmatched.

**Figure 1:**
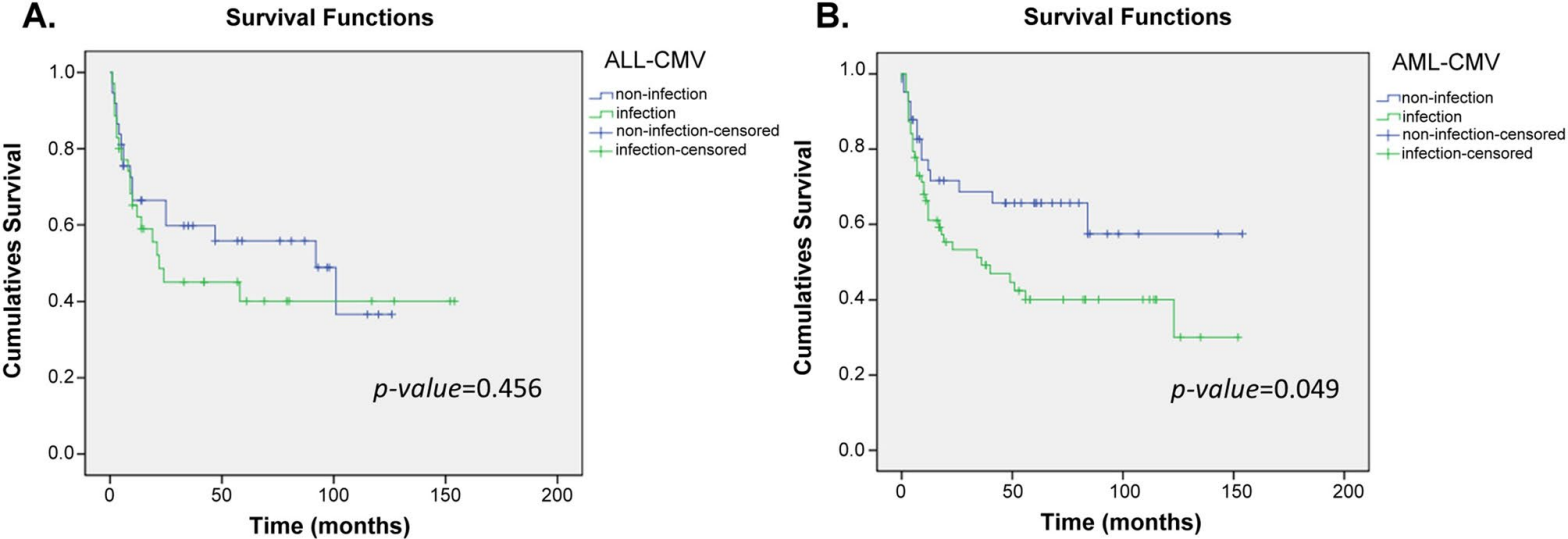
Kaplan–Meier analysis of overall survival curves based on the CMV infection in ALL and AML patients. (**A**) The survivorship curve of CMV in ALL patients. (**B**) The survivorship curve of CMV in AML patients. censoring: the time point of patient death or losing contact; x-axis: the time from receiving transplantation to death. y-axis: the ratio of surviving patients.

**Figure 2:**
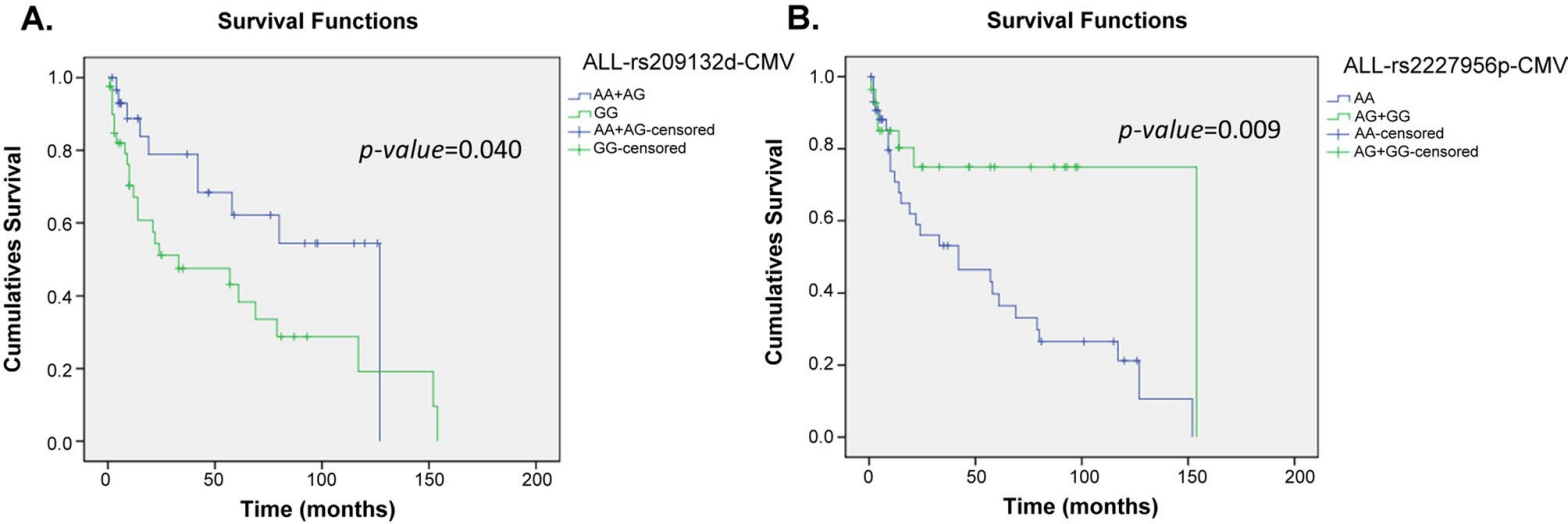
Kaplan–Meier analysis of overall survival curves based on the CMV-related SNPs in ALL patients. (**A**) The rs209132 of TRIM27 in donor group. (**B**) The rs2227956 of HSPA1L in recipient group. censoring: the time point of patient death or losing contact; x-axis: the time from receiving transplantation to death. y-axis: the ratio of surviving patients.

**Figure 3:**
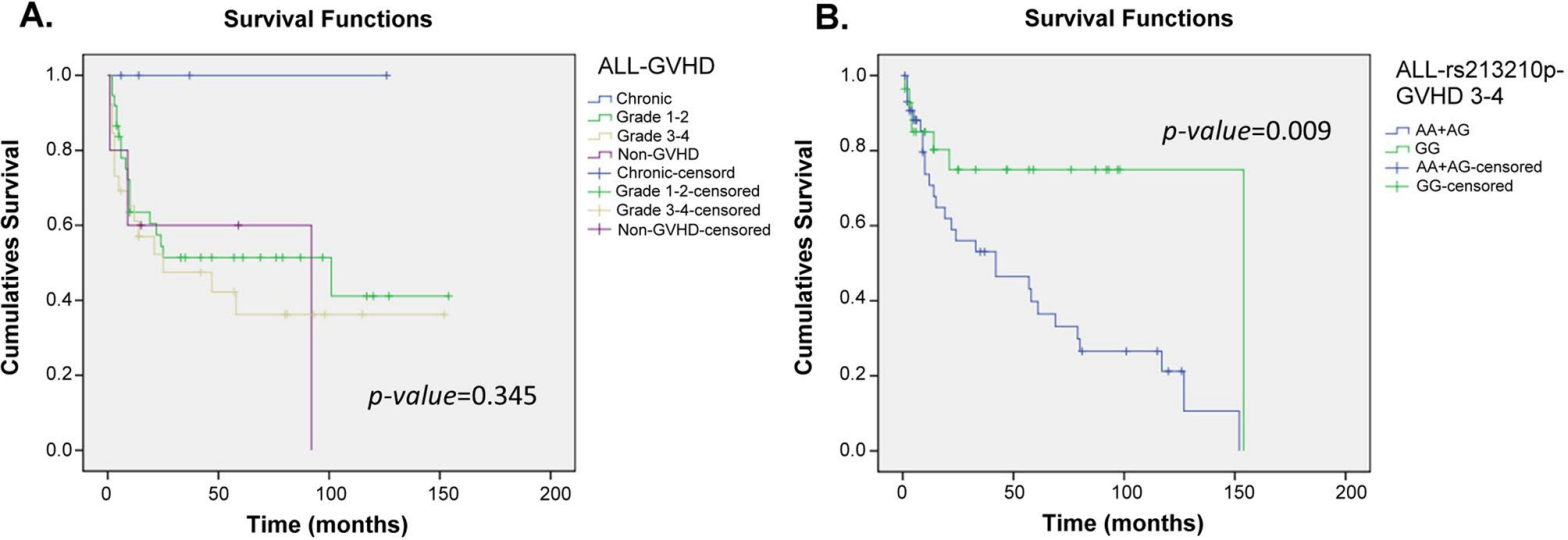
Kaplan–Meier analysis of overall survival curves based on the grade of GVHD and GVHD-related SNPs in ALL patients. (**A**) The survivorship curve of GVHD in ALL patients. (**B**) The rs213210 of RING1 in the recipient group. censoring: the time point of patient death or losing contact; x-axis: the time from receiving transplantation to death. y-axis: the ratio of surviving patients.

**Figure 4:**
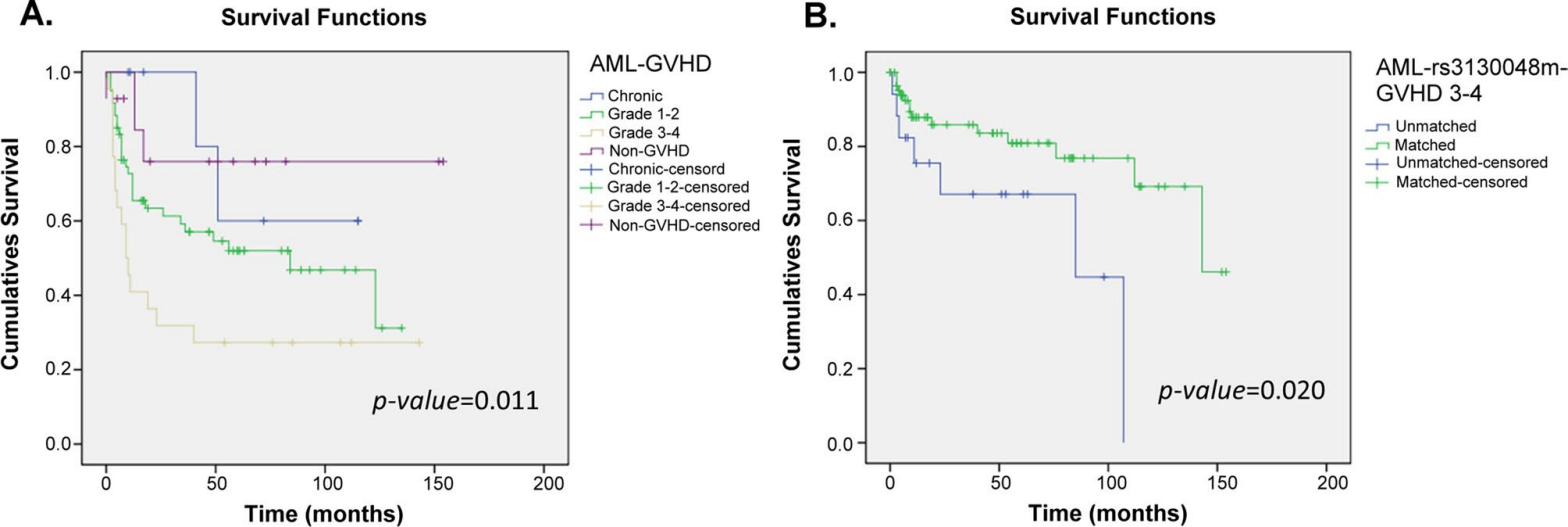
Kaplan–Meier analysis of overall survival curves based on the grade of GVHD and GVHD-related SNPs in AML patients. (**A**) The survivorship curve of GVHD in AML patients. (**B**) The rs3130048 of BAG6 in donor-recipient pairs. censoring: the time point of patient death or losing contact; x-axis: the time from receiving transplantation to death. y-axis: the ratio of surviving patients.

Figures 1, 2, 3 and 4 and S1 to S4 were a follow-up analysis for Tables 2 and 3 to investigate whether CMV infection, severe GVHD, CMV-related SNPs and GVHD-related SNPs would influence overall survival in which the time was calculated from the patient receiving HSCT, and the censoring was indicated the time point of patient death or losing contact. According to the presence or absence of CMV infection, the survival rate was similar in the early stage in ALL and AML groups, and the death toll of CMV infection was gradually increasing after approximately 10 months after transplantation (Fig. 1). However, CMV infection’s overall survival was only significantly correlated in the AML group, p = 0.049 (Fig. 1B). In terms of the survivorship curve of CMV-related SNPs in patients with ALL, the rs209132 of *TRIM27* in the donor group had a significant difference, where patients with minor allele (A allele) had better overall survival, p = 0.040 (Fig. 2A). Furthermore, the G-allele patients in rs2227956 of *HSPA1L* had a better prognosis, p = 0.009 (Fig. 2B).

In ALL group, the highest overall survival was shown in patients with chronic GVHD, and the highest mortality rate was shown in patients with grade 3–4 of GVHD (Fig. 3A). While in the AML group, patients with non-GVHD had the highest survival rate, and the next was chronic GVHD. The highest mortality rate was shown in patients with grade 3–4 of GVHD, and patients with chronic GVHD had continuously survived in the first four years, 48 months (Fig. 4A). Moreover, the grade of GVHD in AML patients was related to the overall survival, p = 0.011 (Fig. 4A). Comparing the results of Table 2 and Fig. 3B, the polymorphism of rs213210 in RING1 was related to grade of GVHD and the overall survival (p = 0.012). Moreover, patients had better overall survival when the genotype of rs3130048 was matched in donor-recipient pairs (Fig. 4B).

For the linkage disequilibrium plot of donor SNPs (Fig. S5), there were 13 SNPs of *HCP5* and *NOTCH4* gene included. One block contained the rs2523676 and rs4713466 with high linkage disequilibrium (LD) (D′ = 0.96) in the *HCP5* gene region and one block contained rs2256594, rs394657 and rs429853 of *NOTCH4* gene with high LD (D′ = 0.97 and D′ = 1). For the LD plot of recipient SNPs (Fig. S6), rs2009658 had a high LD with rs915654 (D′ = 0.92) in the *LTA* gene, rs2075800 had a strong LD with rs2227956 (D′ = 1) in the *HSPA1L* gene and rs9276982 had a high LD with rs79327197 (D′ = 0.83) in the *HLA-DOA* gene. For the LD plot of donor-recipient pairs SNPs (Fig. S7), there were 17 SNPs and three blocks contained in this analysis. The rs209131 and rs209130 of the *TRIM27* gene were contained in block 1. Moreover, two SNP pairs of significant SNPs in the TRIM27 gene had high LD, but they were not considered a block, where rs1536215 had high LD with rs139791445 and rs209130 (D′ = 1). The rs2844464 and the rs2242656 of *BAG6* gene were contained in block 2, while the rs3130048, a statistically significant SNP, had high LD with rs2844464 and rs2242656 (D′ = 0.90), but they were not considered as a block with each SNP. The rs107822 had a high LD with rs213210 (D′ = 0.96) in the *RING1* gene, which both were significant SNPs of SNP analysis, and they were contained together in block 3. Furthermore, the complete data for genetic analysis of the overall SNPs were shown in Tables S1 to S3, except for the outcome-related SNPs.

## Discussion

Our data indicated that 16 candidate SNPs from the nine sourced genes had a significant statistical correlation with HSCT’s adverse reactions. Among these 16 SNPs, the *HCP5*, *BAG6* and *HSPA1L* gene each had one SNP, the *RING1* and *HLA-DOA* gene each had two SNPs, the *HLA-DOB* gene had four SNPs and the *TRIM27* gene had five SNPs. Additionally, the presence of CMV infection and severe GVHD would affect the overall survival in AML patients after receiving HSCT.

Among the nine sourced genes, there were seven genes with HSCT outcomes-related SNPs in our study, except *LTA* and *NOTCH4*. The function of these genes is associated with immunity. HCP5 (HLA complex P5) is a long non-coding RNA (lncRNA) located in the HLA I region, which involved innate and adaptive immune response and association with the occurrence of certain autoimmune diseases and cancer^12^. The rs2518028 was associated with survival rate in AML patients. Moreover, we discovered that the genotype of rs2518028 was related to the risk of relapse in the correlation study of unrelated CBT^10^. HLA-DO is a heterodimer with an alpha and beta chain, and it is a highly conserved molecule^13^. Compared with other HLA II molecules, HLA-DO rarely shows genetic sequence variation, especially at the protein level^14^. The expression of the HLA-DO molecule could ensure the normal development of CD4 memory T cells^15^. Additionally, more and more evidence showed that CD4 + T cells played an important role in regulating CMV infection, reactivation and vertical transmission^16^. The rs79327197 of HLA-DOA, the rs1721369 of HLA-DOB and the rs2070120 of HLA-DOB were found and they were related to relapse in our previous HSCT study^11^. Regarding HSPA1L, which encodes 70 kDa heat shock protein (HSP70) in the HLA class III region^17^. Hsp70 plays a role in cell apoptosis inhibition, and it participates in immune regulation by regulating protein degradation and antigen presentation^18^. Moreover, BAG6 regulates the activity of T cells^19^ and NK cells^20^, and it decreases the expression of MHC class II on antigen-presenting cells^21^. RING1 is in the HLA class II region, which can play a transcriptional repressor role and influence the expression of gene^22^. Notably, the rs107822 and rs213210 in the *RING1* gene region had high LD, and they were considered a haplotype block. It was meant that one of these SNPs with statistical significance might be due to LD with the other. That was necessary to verify further that which SNP was a risk SNP. And TRIM27 is a member of the TRIM protein family, which are widely involved in biological development, including cell proliferation, differentiation, development, morphological changes, apoptosis and TRIM expression proteins are regulated by interferons (IFNs)^23^.

These sourced genes were related to cancer and adverse reactions after transplantation^10,11,24–27^. The several SNPs were associated with relapse in previous studies^10,11^ and related to the grade of GVHD, the rate of survival and the risk of CMV infection in this study. This suggested that these SNPs might play an important role in bone marrow transplantation. Depending on the location of the SNP, the effect on gene performance is different. The genetic variation in the promoter and 3′UTR region may influence the expression level of gene^28,29^, while genetic variation in the intron region may affect the alternative splicing of mRNA and production of proteins with impaired function^30,31^. Moreover, gene variation in the exon region may result in transcription code alternation and cause protein structure changes or termination codes. For example, the nucleotide changes from G to A at rs2227956 results in amino acid replacement from Met to Thr, and it induces the property of HSP70 changing from hydrophobicity to hydrophilicity and altering biological function^32^. Furthermore, this change could influence the substrate specificity and chaperone activity of HSP70^33^, and the SNP has a statistical relationship with the disease, which may not be pathogenic but may be due to the strong LD with the pathogenic gene. Therefore, the SNP’s biological function should be explored by functional analysis in the future to confirm the mechanism of adverse reaction of HSCT caused by the SNP.

The presence or absence of CMV infection and GVHD grade were related to overall survival in AML patients. On the contrary, it did not correlate in ALL patients. Cytomegalovirus might mainly target a population of myeloid lineage progenitor cells derived from bone marrow^34^. Thus, the presence or absence of CMV infection was not significant in the ALL group, even though CMV infection was an important cause of non-relapse mortality after allogeneic HSCT^35^. We found that patients had the highest mortality rate in grades 3–4 regardless of both ALL and AML, but a significant difference was only found in the AML group. However, GVHD had been found to affect the overall survival of AML and ALL patients after allo-HSCT in studies^36,37^. That might be due to our limited sample size; hence, there was no statistical significance in ALL groups. Most of these SNPs were only associated with HSCT outcomes and not related to overall survival in the survival curve of CMV-related SNPs and GVHD-related SNPs. It meant that although these SNPs were associated with the risk of CMV infection or the grade of GVHD, these SNPs were not the major factors affecting the survival time of patients. Only four HSCT-outcome-related SNPs (rs209132, rs2227956, rs3130048 and rs213210) had correlation with overall survival. This suggested that these SNPs might play a vital role in GVHD or CMV infection, which affects the patient’s survival.

Although CBT is one of HSCT, it has a less rigorous requirement for HLA compatibility than bone marrow and peripheral blood for HSCT due to its immunological immaturity; therefore, CBT was not included in this study. We would further explore the correlation between CBT adverse reactions and HLA-related gene SNPs. In conclusion, the issue regarding the correlation between the SNPs of non-HLA genes and the effectiveness of HSCT has continuously been revisited. In this study, we found that several SNPs were related to HSCT’s adverse reactions, and several of them were associated with overall survival. However, these SNPs’ biological function needed to be further investigated to confirm that they directly affect the subsequent gene expression or protein function and then leads to poor prognosis.

## Materials and methods

### Patients and laboratory tests

The Institutional Review Board of Chang Gung Memorial Hospital has reviewed and approved the study. The approval ID was 201304949B0; all study subjects signed informed consent and performed according to the ethical requirement and regulation. A total of 176 patients participated in this study, consisting of 104 patients with AML and 72 patients with ALL who were receiving HSCT in Chang Gung Memorial Hospital. The informed consent forms were written from all enrolled patients. Before transplantation, HLA-A, -B, -C -DRB1 and DQB1 alleles in donor and recipient must be typed by using LABType SSO Typing Test (Thermo Fisher, Waltham, MA) through sequence-specific oligonucleotide probes-based method. Then, the high-resolution HLA-typing was used via the SeCore kit (Thermo Fisher, Waltham, MA) to acquire more detailed allele information. To resolve allele ambiguity from the SeCore typing, the Micro SSP Allele Specific Typing Tray (Thermo Fisher, Waltham, MA) was used by the sequence-specific primers-based method. The characteristics of the patient were shown in Table 1.

### Assessment of adverse reaction after HSCT

Survival was defined as the state of the patient who was still alive at the end of this study. The GVHD was divided into acute GVHD and chronic GVHD, which was based on the occurrence of GVHD within or over 100 days after transplantation^38^, and the acute GVHD was classified into four grades (1 to 4) given organ clinical features according to the National Institutes of Health Consensus. The CMV infection was defined as the presence of CMV antigens or CMV DNA on the recipient’s leukocytes or in recipient’s DNA after transplantation. We only focused on CMV infection after HSCT, no matter it was the first infection or reactivation. The monoclonal antibodies were used to detect viral pp56 antigen on peripheral blood leukocytes, and the CMV DNA was detected by quantitative PCR through whole blood.

### Selection of SNPs

These nine sourced SNPs originated from the literature written by Petersdorf^9^, which showed that these SNPs were associated with the risk of relapse, GVHD, CMV infection and survival rate. A total of 41 SNPs was selected to be candidates from 500 bp upstream and downstream of those nine sourced SNPs. These selected SNPs were also divided into three groups, according to Petersdorf’s study. In other words, we focused on the relationship between the adverse reactions and the SNP in donors if it was showed that the donors with the SNP were related to the effectiveness of allogeneic HSCT in Petersdorf’s study, and this SNP would be distinguished into ‘donor group.’ The classification criteria of the other two groups were similar (Table 4).

**Table 4:** The candidate SNPs that are within 500 bps upstream or downstream of the sourced SNPs.

Gene	Sourced SNP	Model	candidate SNP under analysis						
HCP5	rs2244546	Donor	rs9281491	rs2244546	rs4713466	rs2523676	rs2523675	rs2518028	rs1414315
NOTCH4	rs394657	Donor	rs111394117	rs429853	rs394657	rs2256594	rs444472	rs61365987	
HLA-DOA	rs429916	Recipient	rs9276982	rs71565361	rs79327197	rs151190962	rs9282369		
LTA	rs915654	Recipient	rs2009658	rs736160	rs915654				
HSPA1L	rs2075800	Recipient	rs34324979	rs2075800	rs2227956				
BAG6	rs2242656	Mismatch	rs3130048	rs2844464	rs2242656				
RING1	rs107822	Mismatch	rs107822	rs213210					
TRIM27	rs209130	Mismatch	rs209132	rs209131	rs209130	rs1536215	rs139791445		
HLA-DOB	rs2071479	Mismatch	rs11244	rs2070120	rs56150445	rs41258084	rs17220087	rs2071479	rs17213693

### PCR and sequencing

The genomic DNA was extracted via the QIAamp DNA Blood mini kit (Qiagen, Valencia, CA) from a peripheral blood sample. The purpose was to look for genetic variation within 500 base pairs downstream and upstream of these nine sourced genes. A 50 μl volume of PCR mixture contains 1× reaction buffer, 10 nmol of dNTP, 6 pmol of forward and reversed primers, 300 ng of genomic DNA and 1 μl of Pfu Turbo Hotstart DNA Polymerase (Agilent, Santa Clara, CA). PCR programme was 1 cycle of 94 °C for 4 min, 30 cycles of 94 °C for 30 s, 58 °C for 30 s and 72 °C for 45 s, and the last one cycle was 72 °C for 10 min. After PCR was finished, 5 μl of PCR products were pipetted onto a 2% agarose gel and visualised under UV illumination. The remaining PCR product was directly sequenced by the Big Dye Terminator Cycle Sequencing kit (Thermo Fisher, Waltham, MA) and an ABI PRISM Genetic Analyzer (Thermo Fisher, Waltham, MA) according to the instruction of the manufacturer.

### Statistical analysis

The 41 SNPs in Table 4 were divided into the donor, recipient and donor-recipient pairs groups, and which were analysed with the survival rate, GVHD and CMV infection in both AML and ALL groups. The SPSS (SPSS Inc. Released 2008. SPSS Statistics for Windows, Version 17.0. Chicago, USA) was used to analyse genotype frequency and survival curve. Genotype frequency was analysed using Chi-square and Fisher’s exact tests through a genetic model (dominant, recessive and additive model), in which the allele with higher frequency in ALL or AML population was defined as a major allele, and the lower was minor allele. The survival curve was analysed via Kaplan–Meier analysis and log-rank test. D′ is the normalised standard measurement of LD by contrasting the observed and expected frequencies of one haplotype involved by alleles at different loci. The block was defined as it scarcely had evidence for historical recombination in this region (Gabriel SB, Science, 2002). Haploview 4.2 (https://www.broadinstitute.org/haploview/haploview) was used to analyse LD for the SNPs in donor, recipient and donor-recipient pairs groups, which was used to measure the non-random association of two or more loci in the general population. The blocks were produced by the default algorithm taken from Gabriel et al., Science, 2002.

## Supplementary Information

Supplementary Information
