# Supplementary material for: The adverse events of haematopoietic stem cell transplantation are associated with gene polymorphism within human leukocyte antigen region
Source: Sci Rep. 2021 Jan 14;11:1475. doi: 10.1038/s41598-020-79369-w (PMC7809291; doi:10.1038/s41598-020-79369-w)
Supplement: Supplementary file 1 — Supplementary Information [file 41598_2020_79369_MOESM1_ESM.doc]

**The adverse events of hematopoietic stem cell transplantation are associated with gene polymorphism within HLA region**

Ding-Ping Chen^1,2,3^, Ying-Hao Wen^1,4^, Po-Nan Wang^5^, Ai-Ling Hour^6^, Wei-Tzu Lin^1^, Fang-Ping Hsu^1^, Wei-Ting Wang^1^

^1^ Department of Laboratory Medicine, Chang Gung Memorial Hospital, Taoyuan County, Taiwan

^2^ Department of Medical Biotechnology and Laboratory Science, College of Medicine, Chang Gung University, Taoyuan County, Taiwan

^3^ Graduate Institute of Biomedical Sciences, College of Medicine, Chang Gung University, Taoyuan County, Taiwan

^4^ Graduate Institute of Clinical Medical Sciences, College of Medicine, Chang Gung University, Taoyuan, Taiwan.

^5^ Division of Hematology-Oncology, Department of Internal Medicine, Chang Gung Memorial Hospital, Taoyuan, Taiwan

^6^ Department of Life Science, Fu Jen Catholic University, Taipei, Taiwan.

Figure S1. Kaplan–Meier analysis of overall survival curves based on the CMV infection and CMV-related SNPs in ALL patients. (A)The rs3130048 of BAG6 in recipient group. (B)The rs9282369 of HLA-DOA in recipient group. censoring: the time point of patient death or losing contact; x-axis: the time from receiving transplantation to death. y-axis: the ratio of surviving patients.

Figure S2. Kaplan–Meier analysis of overall survival curves based on the CMV infection and CMV-related SNPs in AML patients. (A)The rs11244 of HLA-DOB in donor group. (B) The rs209131 of TRIM27 in donor group. (C) The rs209131 of TRIM27 in recipient group. (D) The rs209132 of TRIM27 in recipient group. (E) The rs2070120 of HLA-DOB in recipient group. (F) The rs17213693 of HLA-DOB in recipient group. (G) The rs209131 of in donor-recipient pairs. (H)The rs17213693 of HLA-DOB in donor-recipient pairs. censoring: the time point of patient death or losing contact; x-axis: the time from receiving transplantation to death. y-axis: the ratio of surviving patients.


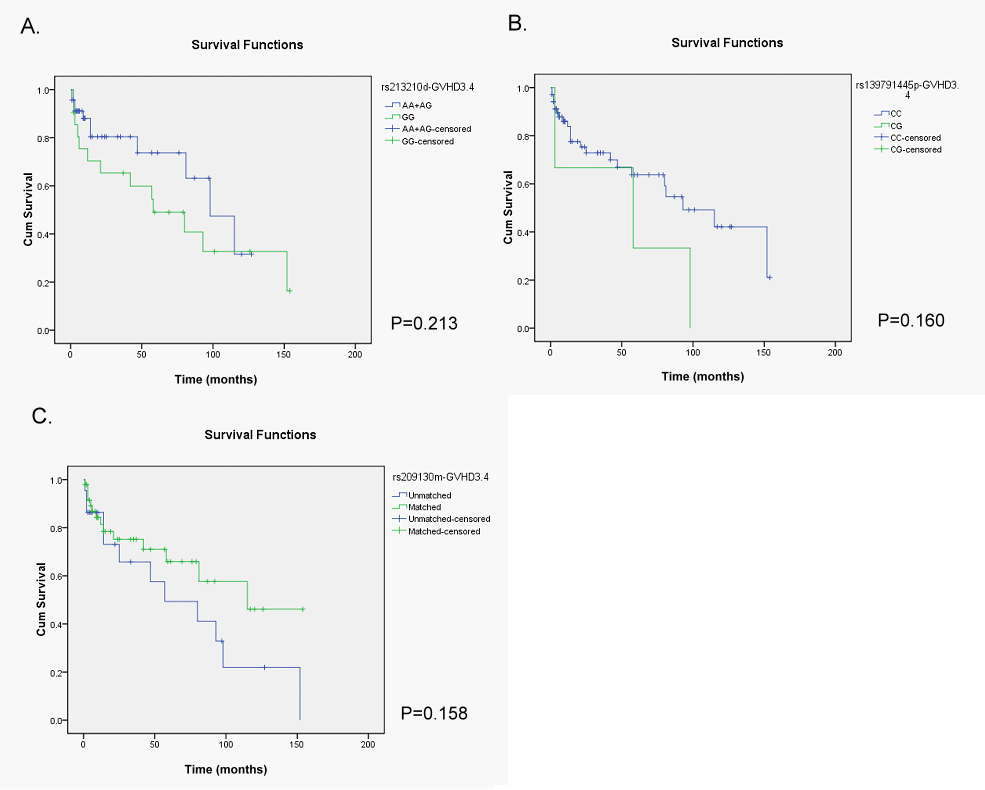


Figure S3. Kaplan–Meier analysis of overall survival curves based on the GVHD-related SNPs in ALL patients. (A)The rs213210 of RING1 in donor group. (B). The rs139791445 of TRIM27 in recipient group. (C) The rs209130 of TRIM27 in donor-recipient pairs. censoring: the time point of patient death or losing contact; x-axis: the time from receiving transplantation to death. y-axis: the ratio of surviving patients.

Figure 4. The survival after HSCT according to grade of GVHD and GVHD -related genotypes in AML patients. The rs1536215 of TRIM27 in donor group. censoring: the time point of patient death or losing contact; x-axis: the time from receiving transplantation to death. y-axis: the ratio of surviving patients.


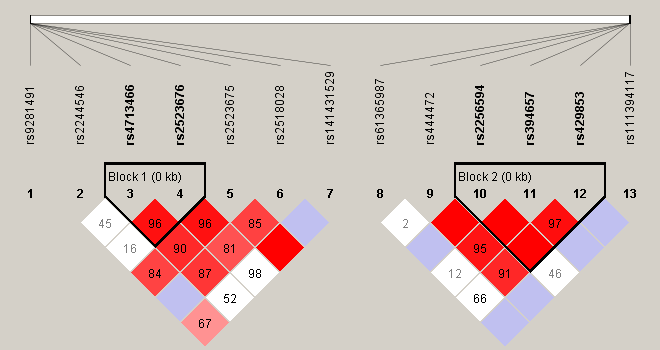


Figure S5. The linkage disequilibrium (LD) analysis between the selected SNPs in the donor group. The D’ measures between the pair of 13 SNPs on NOTCH4 and HCP5 were calculated using the software HaploView 4.2. The red color in the boxes means the two SNPs have high linkage; the closer to white color means that the linkage decreases gradually; the light purple color means they absolutely have no linkage.


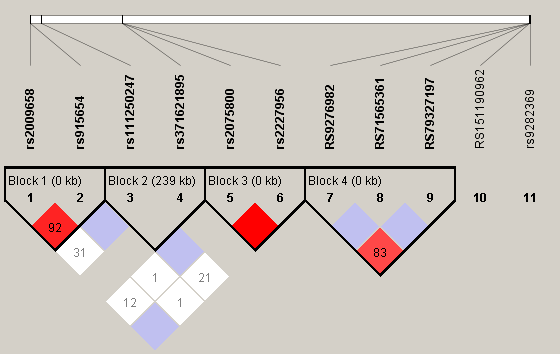


Figure S6. The linkage disequilibrium (LD) analysis between the selected SNPs in the recipient group. The D’ measures between the pairs of 11 SNPs on, HLA-DOA, LTA and HSPA1L were calculated using the software HaploView 4.2. The red color in the boxes means the two SNPs have high linkage; the closer to white color means that the linkage decreases gradually; the light purple color means they absolutely have no linkage.


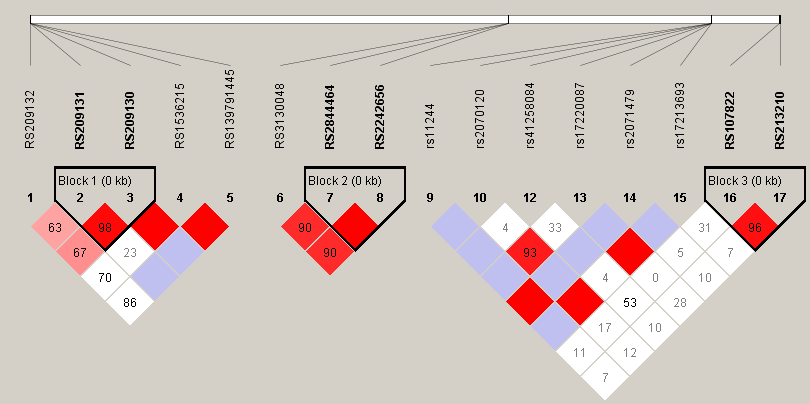


Figure S7. The linkage disequilibrium (LD) analysis between the selected SNPs in the donor-recipient pairs group. The D’ measures between the pair of 17 SNPs on BAG6, HLA-DOB, RING1, and TRIM27 were calculated using the software HaploView 4.2. The red color in the boxes means the two SNPs have high linkage; the closer to white color means that the linkage decreases gradually; the light purple color means they absolutely have no linkage.

Table S1. The complete data for genetic analysis of SNPs in donor group, except for the BMT-related SNPs.

| **SNP** | **Gene** | **Chromosome position (bp)** | **Source** | **Outcome /status** | **Recipient Genotype frequency (%)** | | | **Test** | ***p*-value** | **OR (95% CI)** |
| --- | --- | --- | --- | --- | --- | --- | --- | --- | --- | --- |
| ALL patients | | | | | | | | | | |
| rs9281491 | HCP5 | Chr6: 31435815:31435816 | rs2244546 | CMV | A/A | A/- | -/- |  |  |  |
|  | ~2.5k downstream |  |  | Yes | 6 | 7 | 24 | D | 0.131 | 2.077 (0.800-5.389) |
|  |  |  |  | No | 2 | 16 | 16 |  |  |  |
|  |  |  |  | Survival |  |  |  |  |  |  |
|  |  |  |  | Yes | 2 | 11 | 23 | R | 0.151 | 0.284 (0.053-1.518) |
|  |  |  |  | No | 6 | 12 | 17 |  |  |  |
|  |  |  |  | GVHD3-4 |  |  |  |  |  |  |
|  |  |  |  | Yes | 3 | 7 | 15 | D | 0.646 | 0.794 (0.295-2.132) |
|  |  |  |  | No | 5 | 16 | 25 |  |  |  |
| rs2244546 | HCP5 | Chr6: 31468056 | rs2244546 | CMV | C/C | C/G | G/G |  |  |  |
|  | ~2.5k downstream |  |  | Yes | 32 | 3 | . | D/A | 0.711 | 0.600 (0.132-2.724) |
|  |  |  |  | No | 32 | 5 | . |  |  |  |
|  |  |  |  | Survival |  |  |  |  |  |  |
|  |  |  |  | Yes | 32 | 4 | . | D/A | 1.000 | 1.000 (0.230-4.349) |
|  |  |  |  | No | 32 | 4 | . |  |  |  |
|  |  |  |  | GVHD3-4 |  |  |  |  |  |  |
|  |  |  |  | Yes | 23 | 3 | . | D/A | 1.000 | 1.070 (0.234-4.889) |
|  |  |  |  | No | 41 | 5 | . |  |  |  |
| rs4713466 | HCP5 | Chr6: 31435869 | rs2244546 | CMV | C/C | C/T | T/T |  |  |  |
|  | ~2.5k downstream |  |  | Yes | 21 | 10 | 3 | D | 0.511 | 0.728 (0.283-1.877) |
|  |  |  |  | No | 20 | 15 | 2 |  |  |  |
|  |  |  |  | Survival |  |  |  |  |  |  |
|  |  |  |  | Yes | 22 | 12 | 2 | D | 0.561 | 0.756 (0.294-1.942) |
|  |  |  |  | No | 19 | 13 | 3 |  |  |  |
|  |  |  |  | GVHD3-4 |  |  |  |  |  |  |
|  |  |  |  | Yes | 14 | 8 | 3 | R | 0.337 | 3.000 (0.467-19.290) |
|  |  |  |  | No | 27 | 17 | 2 |  |  |  |
| rs2523676 | HCP5 | Chr6: 31468214 | rs2244546 | CMV | C/C | C/T | T/T |  |  |  |
|  | ~2.5k downstream |  |  | Yes | 19 | 12 | 3 | D | 0.542 | 0.748 (0.294-1.905) |
|  |  |  |  | No | 18 | 16 | 3 |  |  |  |
|  |  |  |  | Survival |  |  |  |  |  |  |
|  |  |  |  | Yes | 19 | 14 | 2 | R | 0.429 | 0.456 (0.078-2.662) |
|  |  |  |  | No | 18 | 13 | 4 |  |  |  |
|  |  |  |  | GVHD3-4 |  |  |  |  |  |  |
|  |  |  |  | Yes | 13 | 8 | 4 | R | 0.175 | 4.190 (0.710-24.730) |
|  |  |  |  | No | 24 | 20 | 2 |  |  |  |
| rs2523675 | HCP5 | Chr6: 31468255 | rs2244546 | CMV | G/G | A/G | A/A |  |  |  |
|  | ~2.5k downstream |  |  | Yes | 7 | 18 | 9 | D | 0.059 | 2.727 (0.948-7.846) |
|  |  |  |  | No | 15 | 12 | 10 |  |  |  |
|  |  |  |  | Survival |  |  |  |  |  |  |
|  |  |  |  | Yes | 11 | 16 | 9 | R | 0.599 | 0.758 (0.269-2.133) |
|  |  |  |  | No | 11 | 14 | 11 |  |  |  |
|  |  |  |  | GVHD3-4 |  |  |  |  |  |  |
|  |  |  |  | Yes | 8 | 11 | 7 | R | 0.903 | 0.935 (0.318-2.749) |
|  |  |  |  | No | 14 | 19 | 13 |  |  |  |
| rs2518028 | HCP5 | Chr6: 31468270 | rs2244546 | CMV | C/C | C/T | T/T |  |  |  |
|  | ~2.5k downstream |  |  | Yes | 28 | 5 | 2 | D | 0.341 | 0.5913 (0.199-1.753) |
|  |  |  |  | No | 26 | 9 | 2 |  |  |  |
|  |  |  |  | Survival |  |  |  |  |  |  |
|  |  |  |  | Yes | 25 | 8 | 3 | D | 0.276 | 1.823 (0.614-5.412) |
|  |  |  |  | No | 29 | 6 | 1 |  |  |  |
|  |  |  |  | GVHD3-4 |  |  |  |  |  |  |
|  |  |  |  | Yes | 21 | 4 | 1 | D | 0.395 | 0.604 (0.188-1.943) |
|  |  |  |  | No | 33 | 10 | 3 |  |  |  |
| rs141431529 | HCP5 | Chr6: 31468278 | rs2244546 | CMV | G/G | G/T | T/T |  |  |  |
|  | ~2.5k downstream |  |  | Yes | 32 | 3 | . | D/A | 1.000 | 1.063 (0.200-5.653) |
|  |  |  |  | No | 34 | 3 | . |  |  |  |
|  |  |  |  | Survival |  |  |  |  |  |  |
|  |  |  |  | Yes | 34 | 2 | . | D/A | 0.674 | 0.471 (0.081-2.748) |
|  |  |  |  | No | 32 | 4 | . |  |  |  |
|  |  |  |  | GVHD3-4 |  |  |  |  |  |  |
|  |  |  |  | Yes | 25 | 1 | . | D/A | 0.408 | 0.328 (0.036-2.972) |
|  |  |  |  | No | 41 | 5 | . |  |  |  |
| rs61365987 | NOTCH4 | Chr: 32218864 | rs394657 | CMV | C/C | C/T | T/T |  |  |  |
|  | intron |  |  | Yes | 31 | 4 | . | D/A | 0.707 | 1.462 (0.303-7.058) |
|  |  |  |  | No | 34 | 3 | . |  |  |  |
|  |  |  |  | Survival |  |  |  |  |  |  |
|  |  |  |  | Yes | 32 | 4 | . | D/A | 1.000 | 1.375 (0.285-6.635) |
|  |  |  |  | No | 33 | 3 | . |  |  |  |
|  |  |  |  | GVHD3-4 |  |  |  |  |  |  |
|  |  |  |  | Yes | 22 | 4 | . | D/A | 0.244 | 2.606 (0.535-12.686) |
|  |  |  |  | No | 43 | 3 | . |  |  |  |
| rs444472 | NOTCH4 | Chr: 32218949 | rs394657 | CMV | G/G | A/G | A/A |  |  |  |
|  | intron |  |  | Yes | 22 | 13 | . | D | 0.148 | 2.142 (0.757-6.064) |
|  |  |  |  | No | 29 | 6 | 2 |  |  |  |
|  |  |  |  | Survival |  |  |  |  |  |  |
|  |  |  |  | Yes | 24 | 10 | 2 | D | 0.437 | 1.500 (0.539-4.178) |
|  |  |  |  | No | 27 | 9 | . |  |  |  |
|  |  |  |  | GVHD3-4 |  |  |  |  |  |  |
|  |  |  |  | Yes | 16 | 9 | 1 | D | 0.192 | 1.989 (0.702-5.631) |
|  |  |  |  | No | 35 | 10 | 1 |  |  |  |
| rs2256594 | NOTCH4 | Chr: 32219095 | rs394657 | CMV | G/G | A/G | A/A |  |  |  |
|  | intron |  |  | Yes | 7 | 13 | 15 | D | 0.502 | 0.722 (0.249-1.869) |
|  |  |  |  | No | 7 | 17 | 13 |  |  |  |
|  |  |  |  | Survival |  |  |  |  |  |  |
|  |  |  |  | Yes | 6 | 13 | 17 | D | 0.147 | 0.492 (0.187-1.291) |
|  |  |  |  | No | 8 | 17 | 11 |  |  |  |
|  |  |  |  | GVHD3-4 |  |  |  |  |  |  |
|  |  |  |  | Yes | 4 | 13 | 9 | R | 0.513 | 0.655 (0.183-2.343) |
|  |  |  |  | No | 10 | 17 | 19 |  |  |  |
| rs394657 | NOTCH4 | Chr: 32219246 | rs394657 | CMV | G/G | A/G | A/A |  |  |  |
|  | intron |  |  | Yes | . | 13 | 22 | D | 0.148 | 2.142 (0.757-6.064) |
|  |  |  |  | No | 2 | 6 | 29 |  |  |  |
|  |  |  |  | Survival |  |  |  |  |  |  |
|  |  |  |  | Yes | 2 | 11 | 23 | D | 0.195 | 1.978 (0.700-5.592) |
|  |  |  |  | No | . | 8 | 28 |  |  |  |
|  |  |  |  | GVHD3-4 |  |  |  |  |  |  |
|  |  |  |  | Yes | 1 | 8 | 17 | D | 0.444 | 1.500 (0.529-4.253) |
|  |  |  |  | No | 1 | 11 | 34 |  |  |  |
| rs429853 | NOTCH4 | Chr: 32219425 | rs394657 | CMV | C/C | C/T | T/T |  |  |  |
|  | intron |  |  | Yes | 0 | 10 | 25 | D | 0.335 | 1.714 (0.569-5.161) |
|  |  |  |  | No | 2 | 5 | 30 |  |  |  |
|  |  |  |  | Survival |  |  |  |  |  |  |
|  |  |  |  | Yes | 2 | 8 | 26 | D | 0.405 | 1.593 (0.530-4.794) |
|  |  |  |  | No | 0 | 7 | 29 |  |  |  |
|  |  |  |  | GVHD3-4 |  |  |  |  |  |  |
|  |  |  |  | Yes | 1 | 7 | 18 | D | 0.282 | 1.827 (0.604-5.525) |
|  |  |  |  | No | 1 | 8 | 37 |  |  |  |
| rs111394117 | NOTCH4 | Chr: 32219436 | rs394657 | CMV | G/G | A/G | A/A |  |  |  |
|  | intron |  |  | Yes | 34 | . | 1 | R | 0.486 | NA |
|  |  |  |  | No | 34 | 3 | . |  |  |  |
|  |  |  |  | Survival |  |  |  |  |  |  |
|  |  |  |  | Yes | 36 | . | . | D | 0.115 | NA |
|  |  |  |  | No | 32 | 3 | 1 |  |  |  |
|  |  |  |  | GVHD3-4 |  |  |  |  |  |  |
|  |  |  |  | Yes | 25 | 1 | . | D | 1.000 | 0.573 (0.057-5.812) |
|  |  |  |  | No | 43 | 2 | 1 |  |  |  |
| rs3130048 | BAG6 | Chr 6: 31645962 | rs2242656 | Survival | C/C | C/T | T/T |  |  |  |
|  | intron |  |  | Yes | 4 | 17 | 14 | D | 0.397 | 1.500 (0.586-3.841) |
|  |  |  |  | No | 3 | 15 | 18 |  |  |  |
|  |  |  |  | GVHD3-4 |  |  |  |  |  |  |
|  |  |  |  | Yes | 2 | 12 | 12 | D | 0.889 | 0.933 (0.354-2.461) |
|  |  |  |  | No | 5 | 20 | 20 |  |  |  |
| rs2844464 | BAG6 | Chr 6: 31646214 | rs2242656 | CMV | A/A | A/G | G/G |  |  |  |
|  | intron |  |  | Yes | 3 | 9 | 23 | D | 0.230 | 1.891 (0.663-5.398) |
|  |  |  |  | No | 2 | 6 | 29 |  |  |  |
|  |  |  |  | Survival |  |  |  |  |  |  |
|  |  |  |  | Yes | 3 | 6 | 27 | D | 0.599 | 0.758 (0.269-2.133) |
|  |  |  |  | No | 2 | 9 | 25 |  |  |  |
|  |  |  |  | GVHD3-4 |  |  |  |  |  |  |
|  |  |  |  | Yes | . | 4 | 22 | D | 0.078 | 0.341 (0.100-1.162) |
|  |  |  |  | No | 5 | 11 | 30 |  |  |  |
| rs2242656 | BAG6 | Chr 6: 31646325 | rs2242656 | CMV | C/C | C/T | T/T |  |  |  |
|  | intron |  |  | Yes | 3 | 7 | 25 | R | 0.670 | 1.641 (0.257-10.458) |
|  |  |  |  | No | 2 | 7 | 28 |  |  |  |
|  |  |  |  | Survival |  |  |  |  |  |  |
|  |  |  |  | Yes | 3 | 6 | 27 | D | 0.789 | 0.867 (0.303-2.475) |
|  |  |  |  | No | 2 | 8 | 26 |  |  |  |
|  |  |  |  | GVHD3-4 |  |  |  |  |  |  |
|  |  |  |  | Yes | . | 4 | 22 | D | 0.111 | 0376 (0.110-1.287) |
|  |  |  |  | No | 5 | 10 | 31 |  |  |  |
| rs107822 | RING1 | Chr 6: 33207798 | rs107822 | CMV | C/C | C/T | T/T |  |  |  |
|  | promoter |  |  | Yes | 5 | 14 | 15 | D | 0.518 | 0.724 (0.272-1.929) |
|  |  |  |  | No | 5 | 16 | 12 |  |  |  |
|  |  |  |  | Survival |  |  |  |  |  |  |
|  |  |  |  | Yes | 5 | 15 | 13 | D | 0.822 | 1.077 (0.405-2.860) |
|  |  |  |  | No | 5 | 15 | 14 |  |  |  |
|  |  |  |  | GVHD3-4 |  |  |  |  |  |  |
|  |  |  |  | Yes | 4 | 7 | 13 | D | 0.084 | 0.408 (0.146-1.139) |
|  |  |  |  | No | 6 | 23 | 14 |  |  |  |
| rs213210 | RING1 | Chr 6:33208047 | rs107822 | CMV | A/A | A/G | G/G |  |  |  |
|  | promoter |  |  | Yes | 9 | 13 | 12 | D | 0.479 | 0.688 (0.243-1.945) |
|  |  |  |  | No | 9 | 15 | 9 |  |  |  |
|  |  |  |  | Survival |  |  |  |  |  |  |
|  |  |  |  | Yes | 1 | 14 | 12 | R | 0.304 | 0.563 (0.187-1.693) |
|  |  |  |  | No | 11 | 114 | 9 |  |  |  |
| rs209132 | TRIM27 | Chr 6: 28899705 | rs209130 | Survival | A/A | A/G | G/G |  |  |  |
|  | ~3k downstream |  |  | Yes | 4 | 15 | 17 | D | 0.069 | 2.439 (0.926-6.421) |
|  |  |  |  | No | 1 | 10 | 24 |  |  |  |
|  |  |  |  | GVHD3-4 |  |  |  |  |  |  |
|  |  |  |  | Yes | . | 11 | 15 | R | 0.151 | NA |
|  |  |  |  | No | 5 | 14 | 26 |  |  |  |
| rs209131 | TRIM27 | Chr 6: 28899978 | rs209130 | CMV | A/A | A/G | G/G |  |  |  |
|  | ~3k downstream |  |  | Yes | 5 | 17 | 13 | D | 0.557 | 0.745 (0.278-1.997) |
|  |  |  |  | No | 5 | 20 | 11 |  |  |  |
|  |  |  |  | Survival |  |  |  |  |  |  |
|  |  |  |  | Yes | 6 | 18 | 12 | R | 0.735 | 1.550 (0.397-6.046) |
|  |  |  |  | No | 4 | 19 | 12 |  |  |  |
|  |  |  |  | GVHD3-4 |  |  |  |  |  |  |
|  |  |  |  | Yes | 3 | 13 | 10 | D | 0.528 | 0.723 (0.263-1.986) |
|  |  |  |  | No | 7 | 24 | 14 |  |  |  |
| rs209130 | TRIM27 | Chr 6: 28900023 | rs209130 | CMV | C/C | C/T | T/T |  |  |  |
|  | ~3k downstream |  |  | Yes | 1 | 13 | 21 | D | 0.190 | 0.533 (0.205-1370) |
|  |  |  |  | No | 3 | 17 | 16 |  |  |  |
|  |  |  |  | Survival |  |  |  |  |  |  |
|  |  |  |  | Yes | 3 | 18 | 15 | D | 0.074 | 2.369 (0.913-6.148) |
|  |  |  |  | No | 1 | 12 | 22 |  |  |  |
|  |  |  |  | GVHD3-4 |  |  |  |  |  |  |
|  |  |  |  | Yes | . | 15 | 11 | D | 0.209 | 1.866 (0.702-4.959) |
|  |  |  |  | No | 4 | 15 | 26 |  |  |  |
| rs1536215 | TRIM27 | Chr 6: 28900138 | rs209130 | CMV | C/C | C/G | G/G |  |  |  |
|  | ~3k downstream |  |  | Yes | 21 | 14 | . | D | 0.177 | 2.000 (0.726-5.508) |
|  |  |  |  | No | 27 | 7 | 2 |  |  |  |
|  |  |  |  | Survival |  |  |  |  |  |  |
|  |  |  |  | Yes | 28 | 8 | . | D | 0.063 | 0.381 (0.136-1.069) |
|  |  |  |  | No | 20 | 13 | 2 |  |  |  |
|  |  |  |  | GVHD3-4 |  |  |  |  |  |  |
|  |  |  |  | Yes | 20 | 6 | . | D | 0.202 | 0.494 (0.166-1.475) |
|  |  |  |  | No | 28 | 15 | 2 |  |  |  |
| rs139791445 | TRIM27 | Chr 6: 28900314 | rs209130 | CMV | C/C | C/G | G/G |  |  |  |
|  | ~3k downstream |  |  | Yes | 33 | 2 | . | D/A | 1.000 | 0.667 (0.104-4.253) |
|  |  |  |  | No | 33 | 3 | . |  |  |  |
|  |  |  |  | Survival |  |  |  |  |  |  |
|  |  |  |  | Yes | 34 | 2 | . | D/A | 0.674 | 0.627 (0.098-4.003) |
|  |  |  |  | No | 32 | 3 | . |  |  |  |
|  |  |  |  | GVHD3-4 |  |  |  |  |  |  |
|  |  |  |  | Yes | 24 | 2 | . | D/A | 1.000 | 1.167 (0.182-7.481) |
|  |  |  |  | No | 42 | 3 | . |  |  |  |
| rs11244 | HLA-DOB | Chr 6: 32812947 | rs2071479 | CMV | A/A | A/G | G/G |  |  |  |
|  | 3’UTR |  |  | Yes | 3 | 11 | 21 | R | 0.350 | 3.375 (0.334-34.094) |
|  |  |  |  | No | 1 | 15 | 21 |  |  |  |
|  |  |  |  | Survival |  |  |  |  |  |  |
|  |  |  |  | Yes | 1 | 15 | 20 | R | 0.641 | 0.314 (0.031-3.174) |
|  |  |  |  | No | 3 | 11 | 22 |  |  |  |
|  |  |  |  | GVHD3-4 |  |  |  |  |  |  |
|  |  |  |  | Yes | . | 10 | 16 | R | 0.289 | NA |
|  |  |  |  | No | 4 | 16 | 26 |  |  |  |
| rs2070120 | HLA-DOB | Chr 6: 32813137 | rs2071479 | CMV | G/G | A/G | A/A |  |  |  |
|  | 3’UTR |  |  | Yes | 31 | 4 | . | D/A | 0.736 | 1.500 (0.385-5.842) |
|  |  |  |  | No | 31 | 6 | . |  |  |  |
|  |  |  |  | Survival |  |  |  |  |  |  |
|  |  |  |  | Yes | 32 | 4 | . | D/A | 0.735 | 1.600 (0.411-6.232) |
|  |  |  |  | No | 30 | 6 | . |  |  |  |
|  |  |  |  | GVHD3-4 |  |  |  |  |  |  |
|  |  |  |  | Yes | 23 | 3 | . | D/A | 0.739 | 1.376 (0.324-5.851) |
|  |  |  |  | No | 39 | 7 | . |  |  |  |
| rs56150445 | HLA-DOB | Chr 6: 32813158-  32813160 | rs2071479 | CMV | G/G | G/- | -/- |  |  |  |
|  | 3’UTR |  |  | Yes | 34 | 1 | . | D/A | 0.486 | NA |
|  |  |  |  | No | 37 | . | . |  |  |  |
|  |  |  |  | Survival |  |  |  |  |  |  |
|  |  |  |  | Yes | 36 | . | . | D/A | 1.000 | NA |
|  |  |  |  | No | 35 | 1 | . |  |  |  |
|  |  |  |  | GVHD3-4 |  |  |  |  |  |  |
|  |  |  |  | Yes | 26 | . | . | D/A | 1.000 | NA |
|  |  |  |  | No | 45 | 1 | . |  |  |  |
| rs41258084 | HLA-DOB | Chr 6: 32813180 | rs2071479 | CMV | C/C | C/T | T/T |  |  |  |
|  | 3’UTR |  |  | Yes | 29 | 6 | . | D/A | 1.000 | 1.069 (0.309-3.693) |
|  |  |  |  | No | 31 | 6 | . |  |  |  |
|  |  |  |  | Survival |  |  |  |  |  |  |
|  |  |  |  | Yes | 29 | 7 | . | D/A | 0.527 | 1.497 (0.427-5.246) |
|  |  |  |  | No | 31 | 5 | . |  |  |  |
|  |  |  |  | GVHD3-4 |  |  |  |  |  |  |
|  |  |  |  | Yes | 20 | 6 | . | D/A | 0.330 | 2.000 (0.572-6.998) |
|  |  |  |  | No | 40 | 6 | . |  |  |  |
| rs17220087 | HLA-DOB | Chr 6: 32813299 | rs2071479 | CMV | C/C | A/C | A/A |  |  |  |
|  | intron |  |  | Yes | 30 | 4 | 1. | R | 0.486 | NA |
|  |  |  |  | No | 31 | 6 | . |  |  |  |
|  |  |  |  | Survival |  |  |  |  |  |  |
|  |  |  |  | Yes | 32 | 4 | . | D | 0.326 | 0.518 (0.137-1.953) |
|  |  |  |  | No | 29 | 6 | 1 |  |  |  |
|  |  |  |  | GVHD3-4 |  |  |  |  |  |  |
|  |  |  |  | Yes | 23 | 3 | . | D | 0.735 | 0.620 (0.149-2.574) |
|  |  |  |  | No | 38 | 7 | 1 |  |  |  |
| rs2071479 | HLA-DOB | Chr 6: 32813335 | rs2071479 | CMV | C/C | C/T | T/T |  |  |  |
|  | intron |  |  | Yes | 35 | . | . | D/A | 0.240 | NA |
|  |  |  |  | No | 34 | 3 | . |  |  |  |
|  |  |  |  | Survival |  |  |  |  |  |  |
|  |  |  |  | Yes | 36 | . | . | D/A | 0.239 | NA |
|  |  |  |  | No | 33 | 3 | . |  |  |  |
|  |  |  |  | GVHD3-4 |  |  |  |  |  |  |
|  |  |  |  | Yes | 25 | 1 | . | D/A | 1.000 | 0.880 (0.076-10.199) |
|  |  |  |  | No | 44 | 2 | . |  |  |  |
| rs17213693 | HLA-DOB | Chr 6: 32813344 | rs2071479 | CMV | G/G | C/G | C/C |  |  |  |
|  | intron |  |  | Yes | 31 | 4 | . | D/A | 0.736 | 1.500 (0.385-5.842) |
|  |  |  |  | No | 31 | 6 | . |  |  |  |
|  |  |  |  | Survival |  |  |  |  |  |  |
|  |  |  |  | Yes | 32 | 4 | . | D/A | 0.496 | 1.600 (0.411-6.232) |
|  |  |  |  | No | 30 | 6 | . |  |  |  |
|  |  |  |  | GVHD3-4 |  |  |  |  |  |  |
|  |  |  |  | Yes | 23 | 3 | . | D/A | 0.739 | 1.376 (0.324-5.851) |
|  |  |  |  | NO | 39 | 7 | . |  |  |  |
| AML recipient | | | | | | | | | | |
| rs9281491 | HCP5 | Chr6: 31435815:31435816 | rs2244546 | CMV | A/A | A/- | -/- |  |  |  |
|  | ~2.5k downstream |  |  | Yes | 3 | 22 | 37 | R | 0.260 | 0.366 (0.082-1.625) |
|  |  |  |  | No | 5 | 14 | 22 |  |  |  |
|  |  |  |  | Survival |  |  |  |  |  |  |
|  |  |  |  | Yes | 5 | 18 | 31 | R | 0.718 | 1.565 (0.354-6.920) |
|  |  |  |  | No | 3 | 18 | 28 |  |  |  |
|  |  |  |  | GVHD3-4 |  |  |  |  |  |  |
|  |  |  |  | Yes | 1 | 9 | 12 | D | 0.770 | 1.152 (0.446-2.973) |
|  |  |  |  | No | 7 | 27 | 47 |  |  |  |
| rs2244546 | HCP5 | Chr6: 31468056 | rs2244546 | CMV | C/C | C/G | G/G |  |  |  |
|  | ~2.5k downstream |  |  | Yes | 53 | 9 | . | R | 0.398 | NA |
|  |  |  |  | No | 34 | 6 | 1 |  |  |  |
|  |  |  |  | Survival |  |  |  |  |  |  |
|  |  |  |  | Yes | 44 | 9 | 1 | D | 0.380 | 1.629 (0.544-4.873) |
|  |  |  |  | No | 43 | 6 | . |  |  |  |
|  |  |  |  | GVHD3-4 |  |  |  |  |  |  |
|  |  |  |  | Yes | 18 | 4 | . | D | 0.743 | 1.278 (0.368-4.437) |
|  |  |  |  | No | 69 | 11 | 1 |  |  |  |
| rs4713466 | HCP5 | Chr6: 31435869 | rs2244546 | CMV | C/C | C/T | T/T |  |  |  |
|  | ~2.5k downstream |  |  | Yes | 41 | 18 | 3 | D | 0.819 | 1.103 (0.475-2.561) |
|  |  |  |  | No | 28 | 11 | 2 |  |  |  |
|  |  |  |  | Survival |  |  |  |  |  |  |
|  |  |  |  | Yes | 36 | 16 | 2 | R | 0.667 | 0.590 (0.094-3.686) |
|  |  |  |  | No | 33 | 13 | 3 |  |  |  |
|  |  |  |  | GVHD3-4 |  |  |  |  |  |  |
|  |  |  |  | Yes | 17 | 4 | 1 | D | 0.247 | 0.527 (0.176-1.578) |
|  |  |  |  | No | 52 | 25 | 4 |  |  |  |
| rs2523676 | HCP5 | Chr6: 31468214 | rs2244546 | CMV | C/C | C/T | T/T |  |  |  |
|  | ~2.5k downstream |  |  | Yes | 39 | 19 | 4 | D | 0.843 | 0.921 (0.409-2.075) |
|  |  |  |  | No | 25 | 13 | 3 |  |  |  |
|  |  |  |  | Survival |  |  |  |  |  |  |
|  |  |  |  | Yes | 32 | 18 | 4 | D | 0.527 | 1.294 (0.581-2.881) |
|  |  |  |  | No | 32 | 14 | 3 |  |  |  |
|  |  |  |  | GVHD3-4 |  |  |  |  |  |  |
|  |  |  |  | Yes | 15 | 6 | 1 | D | 0.510 | 0.715 (0.262-1.946) |
|  |  |  |  | No | 49 | 26 | 6 |  |  |  |
| rs2523675 | HCP5 | Chr6: 31468255 | rs2244546 | CMV | G/G | A/G | A/A |  |  |  |
|  | ~2.5k downstream |  |  | Yes | 25 | 26 | 11 | D | 0.375 | 0.687 (0.299-1.577) |
|  |  |  |  | No | 13 | 19 | 9 |  |  |  |
|  |  |  |  | Survival |  |  |  |  |  |  |
|  |  |  |  | Yes | 20 | 23 | 11 | R | 0.797 | 1.137 (0.426-3.031) |
|  |  |  |  | No | 18 | 22 | 9 |  |  |  |
|  |  |  |  | GVHD3-4 |  |  |  |  |  |  |
|  |  |  |  | Yes | 7 | 10 | 5 | D | 0.578 | 1.329 (0.487-3.622) |
|  |  |  |  | No | 31 | 35 | 15 |  |  |  |
| rs2518028 | HCP5 | Chr6: 31468270 | rs2244546 | CMV | C/C | C/T | T/T |  |  |  |
|  | ~2.5k downstream |  |  | Yes | 40 | 16 | 6 | R | 0.472 | 2.089 (0.401-10.897) |
|  |  |  |  | No | 27 | 12 | 2 |  |  |  |
|  |  |  |  | GVHD3-4 |  |  |  |  |  |  |
|  |  |  |  | Yes | 16 | 4 | 2 | D | 0.394 | 0.638 (0.225-1.805) |
|  |  |  |  | No | 51 | 24 | 6 |  |  |  |
| rs141431529 | HCP5 | Chr6: 31468278 | rs2244546 | CMV | G/G | G/T | T/T |  |  |  |
|  | ~2.5k downstream |  |  | Yes | 55 | 7 | . | D/A | 1.000 | 1.177 (0.322-4.308) |
|  |  |  |  | No | 37 | 4 | . |  |  |  |
|  |  |  |  | Survival |  |  |  |  |  |  |
|  |  |  |  | Yes | 48 | 6 | . | D/A | 0.882 | 1.100 (0.313-3.860) |
|  |  |  |  | No | 44 | 5 | . |  |  |  |
|  |  |  |  | GVHD3-4 |  |  |  |  |  |  |
|  |  |  |  | Yes | 20 | 2 | . | D/A | 1.000 | 0.800 (0.160-4.003) |
|  |  |  |  | No | 72 | 9 | . |  |  |  |
| rs61365987 | NOTCH4 | Chr: 32218864 | rs394657 | CMV | C/C | C/T | T/T |  |  |  |
|  | intron |  |  | Yes | 52 | 9 | . | D/A | 0.084 | 6.577 (0.799-54.134) |
|  |  |  |  | No | 38 | 1 | . |  |  |  |
|  |  |  |  | Survival |  |  |  |  |  |  |
|  |  |  |  | Yes | 46 | 5 | . | D/A | 1.000 | 0.957 (0.259-3.533) |
|  |  |  |  | No | 44 | 5 | . |  |  |  |
|  |  |  |  | GVHD3-4 |  |  |  |  |  |  |
|  |  |  |  | Yes | 20 | 2 | . | D/A | 1.000 | 0.875 (0.172-4.454) |
|  |  |  |  | No | 70 | 8 | . |  |  |  |
| rs444472 | NOTCH4 | Chr: 32218949 | rs394657 | CMV | G/G | A/G | A/A |  |  |  |
|  | intron |  |  | Yes | 42 | 17 | 2 | D | 0.142 | 2.068 (0.775-5.516) |
|  |  |  |  | No | 32 | 7 | . |  |  |  |
|  |  |  |  | Survival |  |  |  |  |  |  |
|  |  |  |  | Yes | 38 | 13 | . | R | 0.238 | NA |
|  |  |  |  | No | 36 | 11 | 2 |  |  |  |
|  |  |  |  | GVHD3-4 |  |  |  |  |  |  |
|  |  |  |  | Yes | 18 | 3 | 1 | D | 0.344 | 0.566 (0.172-1.860) |
|  |  |  |  | No | 56 | 21 | 1 |  |  |  |
| rs2256594 | NOTCH4 | Chr: 32219095 | rs394657 | CMV | G/G | A/G | A/A |  |  |  |
|  | intron |  |  | Yes | 6 | 28 | 27 | R | 0.261 | 0.514 (0.1559-1.662) |
|  |  |  |  | No | 7 | 19 | 14 |  |  |  |
|  |  |  |  | Survival |  |  |  |  |  |  |
|  |  |  |  | Yes | 7 | 28 | 17 | D | 0.096 | 1.976 (0.883-4.426) |
|  |  |  |  | No | 6 | 19 | 24 |  |  |  |
|  |  |  |  | GVHD3-4 |  |  |  |  |  |  |
|  |  |  |  | Yes | 2 | 8 | 12 | D | 0.132 | 0.483 (0.186-1.257) |
|  |  |  |  | No | 11 | 39 | 29 |  |  |  |
| rs394657 | NOTCH4 | Chr: 32219246 | rs394657 | CMV | G/G | A/G | A/A |  |  |  |
|  | intron |  |  | Yes | 2 | 18 | 41 | D | 0.160 | 1.951 (0.761-5.001) |
|  |  |  |  | No | . | 8 | 32 |  |  |  |
|  |  |  |  | Survival |  |  |  |  |  |  |
|  |  |  |  | Yes | . | 14 | 38 | R | 0.233 | NA |
|  |  |  |  | No | 2 | 12 | 35 |  |  |  |
|  |  |  |  | GVHD3-4 |  |  |  |  |  |  |
|  |  |  |  | Yes | 1 | 3 | 18 | D | 0.258 | 0.509 (0.156-1.665) |
|  |  |  |  | No | 1 | 23 | 55 |  |  |  |
| rs429853 | NOTCH4 | Chr: 32219425 | rs394657 | CMV | C/C | C/T | T/T |  |  |  |
|  | intron |  |  | Yes | 1 | 14 | 45 | D | 0.229 | 1.889 (0.663-5.378) |
|  |  |  |  | No | . | 6 | 34 |  |  |  |
|  |  |  |  | Survival |  |  |  |  |  |  |
|  |  |  |  | Yes | . | 10 | 42 | R | 0.480 | NA |
|  |  |  |  | No | 1 | 10 | 37 |  |  |  |
|  |  |  |  | GVHD3-4 |  |  |  |  |  |  |
|  |  |  |  | Yes | . | 2 | 20 | D | 0.148 | 0.311 (0.066-1.452) |
|  |  |  |  | No | 1 | 18 | 59 |  |  |  |
| rs111394117 | NOTCH4 | Chr: 32219436 | rs394657 | CMV | G/G | A/G | A/A |  |  |  |
|  | intron |  |  | Yes | 58 | 1 | 1 | D | 0.513 | NA |
|  |  |  |  | No | 41 | . | . |  |  |  |
|  |  |  |  | Survival |  |  |  |  |  |  |
|  |  |  |  | Yes | 52 | 1 | . | R | 0.475 | NA |
|  |  |  |  | No | 47 | . | 1 |  |  |  |
|  |  |  |  | GVHD3-4 |  |  |  |  |  |  |
|  |  |  |  | Yes | 22 | . | . | D | 1.000 | NA |
|  |  |  |  | No | 77 | 1 | 1 |  |  |  |
| rs107822 | RING1 | Chr 6: 33207798 | rs107822 | CMV | C/C | C/T | T/T |  |  |  |
|  | promoter |  |  | Yes | 9 | 32 | 20 | D | 0.372 | 1.452 (0.640-3.296) |
|  |  |  |  | No | 5 | 19 | 17 |  |  |  |
|  |  |  |  | Survival |  |  |  |  |  |  |
|  |  |  |  | Yes | 8 | 23 | 22 | D | 0.253 | 0.622 (0.275-1.407) |
|  |  |  |  | No | 6 | 28 | 15 |  |  |  |
|  |  |  |  | GVHD3-4 |  |  |  |  |  |  |
|  |  |  |  | Yes | 3 | 8 | 11 | D | 0.131 | 0.481 (0.185-1.255) |
|  |  |  |  | No | 11 | 43 | 26 |  |  |  |
| rs213210 | RING1 | Chr 6:33208047 | rs107822 | CMV | A/A | A/G | G/G |  |  |  |
|  | promoter |  |  | Yes | 17 | 30 | 14 | R | 0.117 | 2.254 (0.804-6.320) |
|  |  |  |  | No | 6 | 22 | 13 |  |  |  |
|  |  |  |  | Survival |  |  |  |  |  |  |
|  |  |  |  | Yes | 10 | 25 | 18 | D | 0.074 | 0.438 (0.174-1.098) |
|  |  |  |  | No | 13 | 27 | 9 |  |  |  |
|  |  |  |  | GVHD3-4 |  |  |  |  |  |  |
|  |  |  |  | Yes | 6 | 9 | 7 | D | 0.521 | 0.714 (0.255-2.001) |
|  |  |  |  | No | 17 | 43 | 20 |  |  |  |
| rs209132 | TRIM27 | Chr 6: 28899705 | rs209130 | CMV | A/A | A/G | G/G |  |  |  |
|  | ~3k downstream |  |  | Yes | 4 | 25 | 33 | D | 0.097 | 0.507 (0.226-1.137) |
|  |  |  |  | No | 3 | 23 | 15 |  |  |  |
|  |  |  |  | Survival |  |  |  |  |  |  |
|  |  |  |  | Yes | 4 | 27 | 24 | D | 0.518 | 1.292 (0.594-2.810) |
|  |  |  |  | No | 3 | 21 | 24 |  |  |  |
|  |  |  |  | GVHD3-4 |  |  |  |  |  |  |
|  |  |  |  | Yes | . | 10 | 12 | R | 0.341 | NA |
|  |  |  |  | No | 7 | 38 | 36 |  |  |  |
| rs209131 | TRIM27 | Chr 6: 28899978 | rs209130 | Survival | A/A | A/G | G/G |  |  |  |
|  | ~3k downstream |  |  | Yes | 13 | 30 | 12 | R | 0.747 | 0.860 (0.345-2.144) |
|  |  |  |  | No | 13 | 24 | 12 |  |  |  |
|  |  |  |  | GVHD3-4 |  |  |  |  |  |  |
|  |  |  |  | Yes | 7 | 12 | 3 | D | 0.237 | 0.459 (0.123-1.708) |
|  |  |  |  | No | 19 | 42 | 21 |  |  |  |
| rs209130 | TRIM27 | Chr 6: 28900023 | rs209130 | CMV | C/C | C/T | T/T |  |  |  |
|  | ~3k downstream |  |  | Yes | 4 | 29 | 30 | D | 0.107 | 0.511 (0.224-1.163) |
|  |  |  |  | No | 3 | 25 | 13 |  |  |  |
|  |  |  |  | Survival |  |  |  |  |  |  |
|  |  |  |  | Yes | 4 | 29 | 22 | D | 0.768 | 1.125 (0.515-2.458) |
|  |  |  |  | No | 3 | 25 | 21 |  |  |  |
|  |  |  |  | GVHD3-4 |  |  |  |  |  |  |
|  |  |  |  | Yes | . | 12 | 10 | R | 0.341 | NA |
|  |  |  |  | No | 7 | 42 | 33 |  |  |  |
| rs1536215 | TRIM27 | Chr 6: 28900138 | rs209130 | CMV | C/C | C/G | G/G |  |  |  |
|  | ~3k downstream |  |  | Yes | 39 | 21 | 2 | D | 0.843 | 0.921 (0.409-2.075) |
|  |  |  |  | No | 25 | 14 | 2 |  |  |  |
|  |  |  |  | Survival |  |  |  |  |  |  |
|  |  |  |  | Yes | 33 | 19 | 3 | D | 0.632 | 1.216 (0.546-2.707) |
|  |  |  |  | No | 31 | 16 | 1 |  |  |  |
| rs139791445 | TRIM27 | Chr 6: 28900314 | rs209130 | CMV | C/C | C/G | G/G |  |  |  |
|  | ~3k downstream |  |  | Yes | 60 | 3 | . | D/A | 1.000 | 0.975 (0.156-6.103) |
|  |  |  |  | No | 39 | 2 | . |  |  |  |
|  |  |  |  | Survival |  |  |  |  |  |  |
|  |  |  |  | Yes | 51 | 4 | . | D/A | 0.367 | 3.765 (0.406-34.888) |
|  |  |  |  | No | 48 | 1 | . |  |  |  |
|  |  |  |  | GVHD3-4 |  |  |  |  |  |  |
|  |  |  |  | Yes | 21 | 1 | . | D/A | 1.000 | 0.929 (0.098-8.754) |
|  |  |  |  | No | 78 | 4 | . |  |  |  |
| rs11244 | HLA-DOB | Chr 6: 32812947 | rs2071479 | Survival | A/A | A/G | G/G |  |  |  |
|  | 3’UTR |  |  | Yes | 2 | 22 | 29 | R | 0.496 | NA |
|  |  |  |  | No | . | 20 | 29 |  |  |  |
|  |  |  |  | GVHD3-4 |  |  |  |  |  |  |
|  |  |  |  | Yes | 1 | 10 | 11 | R | 0.387 | 3.762 (0.226-62.689) |
|  |  |  |  | No | 1 | 32 | 47 |  |  |  |
| rs2070120 | HLA-DOB | Chr 6: 32813137 | rs2071479 | CMV | A/A | A/G | G/G |  |  |  |
|  | 3’UTR |  |  | Yes | . | 11 | 51 | D/A | 0.142 | 2.660 (0.693-10.209) |
|  |  |  |  | No | . | 3 | 37 |  |  |  |
|  |  |  |  | Survival |  |  |  |  |  |  |
|  |  |  |  | Yes | . | 4 | 49 | D/A | 0.059 | 0.318 (0.09.-1.093) |
|  |  |  |  | No | . | 10 | 39 |  |  |  |
|  |  |  |  | GVHD3-4 |  |  |  |  |  |  |
|  |  |  |  | Yes | . | 4 | 18 | D/A | 0.495 | 1.556 (0.437-5.539) |
|  |  |  |  | No | . | 10 | 70 |  |  |  |
| rs56150445 | HLA-DOB | Chr 6: 32813158-  32813160 | rs2071479 | CMV | -/- | -/G | G/G |  |  |  |
|  | 3’UTR |  |  | Yes | . | 1 | 62 | D/A | 1.000 | 1.550 (0.094-25.493) |
|  |  |  |  | No | . | 1 | 40 |  |  |  |
|  |  |  |  | Survival |  |  |  |  |  |  |
|  |  |  |  | Yes | . | 2 | 53 | D/A | 0.497 | NA |
|  |  |  |  | No | . | . | 49 |  |  |  |
|  |  |  |  | GVHD3-4 |  |  |  |  |  |  |
|  |  |  |  | Yes | . | . | 22 | D/A | 1.000 | NA |
|  |  |  |  | No | . | 2 | 80 |  |  |  |
| rs41258084 | HLA-DOB | Chr 6: 32813180 | rs2071479 | CMV | C/C | C/T | T/T |  |  |  |
|  | 3’UTR |  |  | Yes | 55 | 6 | 1 | D | 1.000 | 0.891 (0.262-3.028) |
|  |  |  |  | No | 35 | 5 | . |  |  |  |
|  |  |  |  | Survival |  |  |  |  |  |  |
|  |  |  |  | Yes | 46 | 7 | . | R | 0.480 | NA |
|  |  |  |  | No | 44 | 4 | 1 |  |  |  |
|  |  |  |  | GVHD3-4 |  |  |  |  |  |  |
|  |  |  |  | Yes | 18 | 3 | 1 | R | 0.216 | NA |
|  |  |  |  | No | 72 | 8 | . |  |  |  |
| rs17220087 | HLA-DOB | Chr 6: 32813299 | rs2071479 | CMV | A/A | A/C | C/C |  |  |  |
|  | intron |  |  | Yes | . | 9 | 53 | D/A | 0.357 | 2.094 (0.531-8.262) |
|  |  |  |  | No | . | 3 | 37 |  |  |  |
|  |  |  |  | GVHD3-4 |  |  |  |  |  |  |
|  |  |  |  | Yes | . | 4 | 18 | D/A | 0.284 | 2.000 (0.541-7.388) |
|  |  |  |  | No | . | 8 | 72 |  |  |  |
| rs2071479 | HLA-DOB | Chr 6: 32813335 | rs2071479 | CMV | C/C | C/T | T/T |  |  |  |
|  | intron |  |  | Yes | 60 | 2 | . | D/A | 0.054 | 0.189 (0.036-0.988) |
|  |  |  |  | No | 34 | 6 | . |  |  |  |
|  |  |  |  | Survival |  |  |  |  |  |  |
|  |  |  |  | Yes | 48 | 5 | . | D/A | 0.717 | 1.597 (0.361-7.069) |
|  |  |  |  | No | 46 | 3 | . |  |  |  |
|  |  |  |  | GVHD3-4 |  |  |  |  |  |  |
|  |  |  |  | Yes | 19 | 3 | . | D/A | 0.365 | 2.368 (0.519-10.800) |
|  |  |  |  | No | 75 | 5 | . |  |  |  |
| rs17213693 | HLA-DOB | Chr 6: 32813344 | rs2071479 | CMV | C/C | C/G | G/G |  |  |  |
|  | intron |  |  | Yes | 1 | 9 | 52 | R | 0.202 | 0.422 (0.109-1.638) |
|  |  |  |  | No | . | 3 | 37 |  |  |  |
|  |  |  |  | Survival |  |  |  |  |  |  |
|  |  |  |  | Yes | 1 | 3 | 49 | R | 0.102 | 2.756 (0.790-9.617) |
|  |  |  |  | No | . | 9 | 40 |  |  |  |
|  |  |  |  | GVHD3-4 |  |  |  |  |  |  |
|  |  |  |  | Yes | . | 4 | 18 | D | 0.470 | 0.570 (0.158-2.065) |
|  |  |  |  | NO | 1 | 8 | 71 |  |  |  |

D: dominant model (AA vs. Aa + aa); R: recessive model (AA + Aa vs. aa); A: additive model (AA vs. Aa vs. aa), in which “A” was defined as a higher frequency allele and the lower was “a”.

Table S2. The complete data for genetic analysis of SNPs in recipient group, except for the BMT-related SNPs.

| **SNP** | **Gene** | **Chromosome position (bp)** | **Source** | **Outcome /status** | **Recipient Genotype frequency (%)** | | | **Test** | ***p*-value** | **OR (95% CI)** |
| --- | --- | --- | --- | --- | --- | --- | --- | --- | --- | --- |
| ALL | | | | | | | | | | |
| rs9276982 | HLA-DOA | Chr 6: 33010438 | rs9276982 | CMV | A/A | A/G | G/G |  |  |  |
|  | promoter |  |  | Yes | 1 | 13 | 20 | D | 0.600 | 1.292 (0.495-3.376) |
|  |  |  |  | No | 1 | 12 | 24 |  |  |  |
|  |  |  |  | Survival |  |  |  |  |  |  |
|  |  |  |  | Yes | 0 | 10 | 26 | D | 0.071 | 0.407 (0.152-1.091) |
|  |  |  |  | No | 2 | 15 | 18 |  |  |  |
|  |  |  |  | GVHD3-4 |  |  |  |  |  |  |
|  |  |  |  | Yes | 1 | 7 | 17 | D | 0.440 | 0.669 (0.240-1.864) |
|  |  |  |  | No | 1 | 18 | 27 |  |  |  |
| rs71565361 | HLA-DOA | Chr 6: 33010551 | rs9276982 | CMV | A/A | A/C | C/C |  |  |  |
|  | promoter |  |  | Yes | . | . | 34 | D | 1.000 | NA |
|  |  |  |  | No | . | 1 | 36 |  |  |  |
|  |  |  |  | Survival |  |  |  |  |  |  |
|  |  |  |  | Yes | . | 1 | 35 | D | 1.000 | NA |
|  |  |  |  | No | . | . | 35 |  |  |  |
|  |  |  |  | GVHD3-4 |  |  |  |  |  |  |
|  |  |  |  | Yes | . | . | 25 | D | 1.000 | NA |
|  |  |  |  | No | . | 1 | 45 |  |  |  |
| rs79327197 | HLA-DOA | Chr 6: 33010635 | rs9276982 | CMV | A/A | A/G | G/G |  |  |  |
|  | promoter |  |  | Yes | 29 | 5 | . | D/A | 1.000 | 1.103 (0.290-4.204) |
|  |  |  |  | No | 32 | 5 | . |  |  |  |
|  |  |  |  | Survival |  |  |  |  |  |  |
|  |  |  |  | Yes | 32 | 4 | . | D/A | 0.514 | 1.655 (0.424-6.457) |
|  |  |  |  | No | 29 | 6 | . |  |  |  |
|  |  |  |  | GVHD3-4 |  |  |  |  |  |  |
|  |  |  |  | Yes | 23 | 2 | . | D/A | 0.477 | 0.413 (0.081-2.116) |
|  |  |  |  | No | 38 | 8 | . |  |  |  |
| rs151190962 | HLA-DOA | Chr 6: 33010881 | rs9276982 | CMV | A/A | -/A | -/- |  |  |  |
|  | promoter |  |  | Yes | 34 | . | . | D/A | 1.000 | NA |
|  |  |  |  | No | 36 | 1 | . |  |  |  |
|  |  |  |  | Survival |  |  |  |  |  |  |
|  |  |  |  | Yes | 36 | . | . | D/A | 0.493 | NA |
|  |  |  |  | No | 34 | 1 | . |  |  |  |
|  |  |  |  | GVHD3-4 |  |  |  |  |  |  |
|  |  |  |  | Yes | 25 | . | . | D/A | 1.000 | NA |
|  |  |  |  | No | 45 | 1 | . |  |  |  |
| rs9282369 | HLA-DOA | Chr 6: 33011011-  33011018 | rs9276982 | Survival | -/- | -/T | T/T |  |  |  |
|  | promoter |  |  | Yes | 8 | 20 | 8 | R | 0.112 | 3.048 (0.736-12.615) |
|  |  |  |  | No | 12 | 20 | 3 |  |  |  |
|  |  |  |  | GVHD3-4 |  |  |  |  |  |  |
|  |  |  |  | Yes | 8 | 15 | 2 | R | 0.307 | 0.357 (0.071-1.803) |
|  |  |  |  | No | 12 | 25 | 9 |  |  |  |
| rs2009658 | LTA | Chr 6: 31570467 | rs915654 | CMV | C/C | C/G | G/G |  |  |  |
|  | intron |  |  | Yes | 24 | 10 | . | R | 0.494 | NA |
|  |  |  |  | No | 27 | 8 | 2 |  |  |  |
|  |  |  |  | Survival |  |  |  |  |  |  |
|  |  |  |  | Yes | 28 | 8 | . | R | 0.239 | NA |
|  |  |  |  | No | 23 | 10 | 2 |  |  |  |
|  |  |  |  | GVHD3-4 |  |  |  |  |  |  |
|  |  |  |  | Yes | 21 | 4 | . | D | 0.093 | 0.357 (0.104-1.221) |
|  |  |  |  | No | 30 | 14 | 2 |  |  |  |
| rs111250247 | LTA | Chr 6: 31570719 | rs915654 | CMV | T/T | T/A | A/A |  |  |  |
|  | intron |  |  | Yes | 34 | . | . | NA | NA | NA |
|  |  |  |  | No | 37 | . | . |  |  |  |
|  |  |  |  | Survival |  |  |  |  |  |  |
|  |  |  |  | Yes | 36 | . | . | NA | NA | NA |
|  |  |  |  | No | 35 | . | . |  |  |  |
|  |  |  |  | GVHD3-4 |  |  |  |  |  |  |
|  |  |  |  | Yes | 25 | . | . | NA | NA | NA |
|  |  |  |  | No | 46 | . | . |  |  |  |
| rs915654 | LTA | Chr 6: 31570720 | rs915654 | CMV | A/A | A/T | T/T | D | 0.080 | 0.417 (0.155-1.121) |
|  | intron |  |  | Yes | 16 | 15 | 3 |  |  |  |
|  |  |  |  | No | 10 | 19 | 8 |  |  |  |
|  |  |  |  | Survival |  |  |  |  |  |  |
|  |  |  |  | Yes | 11 | 19 | 6 | D | 0.282 | 1.705 (0.643-4.520) |
|  |  |  |  | No | 15 | 15 | 5 |  |  |  |
|  |  |  |  | GVHD3-4 |  |  |  |  |  |  |
|  |  |  |  | Yes | 9 | 13 | 3 | R | 0.736 | 0.648 (0.155-2.699) |
|  |  |  |  | No | 17 | 21 | 8 |  |  |  |
| rs34324979 | HSPA1L | Chr 6: 31809994-  31809997 | rs2075800 | CMV | -/- | -/G | G/G |  |  |  |
|  | 3’UTR |  |  | Yes | 2 | 6 | 27 | D | 0.303 | 1.896 (0.555-6.482) |
|  |  |  |  | No | 1 | 4 | 32 |  |  |  |
|  |  |  |  | Survival |  |  |  |  |  |  |
|  |  |  |  | Yes | 2 | 6 | 28 | D | 0.358 | 1.771 (0.518-6.052) |
|  |  |  |  | No | 1 | 4 | 31 |  |  |  |
|  |  |  |  | GVHD3-4 |  |  |  |  |  |  |
|  |  |  |  | Yes | 1 | 5 | 20 | D | 0.526 | 1.671 (0.495-5.641) |
|  |  |  |  | No | 2 | 5 | 39 |  |  |  |
| rs2075800 | HSPA1L | Chr 6: 31810169 | rs2075800 | CMV | C/C | C/T | T/T |  |  |  |
|  | exon |  |  | Yes | 15 | 11 | 9 | R | 0.100 | 2.856 (0.790-10.323) |
|  |  |  |  | No | 17 | 16 | 4 |  |  |  |
|  |  |  |  | Survival |  |  |  |  |  |  |
|  |  |  |  | Yes | 18 | 11 | 7 | D | 0.343 | 0.636 (0.250-1.622) |
|  |  |  |  | No | 14 | 16 | 6 |  |  |  |
|  |  |  |  | GVHD3-4 |  |  |  |  |  |  |
|  |  |  |  | Yes | 10 | 10 | 6 | D | 0.442 | 1.467 (0.551-3.905) |
|  |  |  |  | No | 22 | 17 | 7 |  |  |  |
| rs2227956 | HSPA1L | Chr 6: 31810495 | rs2075800 | Survival | A/A | A/G | G/G |  |  |  |
|  | exon |  |  | Yes | 21 | 12 | 3 | D | 0.629 | 1.264 (0.489-3.266) |
|  |  |  |  | No | 23 | 11 | 2 |  |  |  |
|  |  |  |  | GVHD3-4 |  |  |  |  |  |  |
|  |  |  |  | Yes | 16 | 9 | 1 | R | 0.647 | 0.420 (0.044-3.971) |
|  |  |  |  | No | 28 | 14 | 4 |  |  |  |
| rs3130048 | BAG6 | Chr 6: 31645962 | rs2242656 | Survival | C/C | C/T | T/T |  |  |  |
|  | intron |  |  | Yes | 8 | 13 | 15 | R | 0.358 | 1.771 (0.518-6.052) |
|  |  |  |  | No | 5 | 16 | 15 |  |  |  |
|  |  |  |  | GVHD3-4 |  |  |  |  |  |  |
|  |  |  |  | Yes | 3 | 12 | 11 | R | 0.352 | 0.470 (0.117-1.889) |
|  |  |  |  | No | 10 | 17 | 19 |  |  |  |
| rs2844464 | BAG6 | Chr 6: 31646214 | rs2242656 | CMV | A/A | A/G | G/G |  |  |  |
|  | intron |  |  | Yes | 1 | 10 | 24 | D | 0.345 | 1.661 (0.576-4.792) |
|  |  |  |  | No | 2 | 6 | 29 |  |  |  |
|  |  |  |  | Survival |  |  |  |  |  |  |
|  |  |  |  | Yes | 2 | 6 | 28 | D | 0.422 | 0.649 (0.225-1.871) |
|  |  |  |  | No | 1 | 10 | 25 |  |  |  |
|  |  |  |  | GVHD3-4 |  |  |  |  |  |  |
|  |  |  |  | Yes | . | 5 | 21 | D | 0.300 | 0.544 (0.171-1.736) |
|  |  |  |  | No | 3 | 11 | 32 |  |  |  |
| rs2242656 | BAG6 | Chr 6: 31646325 | rs2242656 | CMV | C/C | C/T | T/T |  |  |  |
|  | intron |  |  | Yes | 1 | 10 | 24 | D | 0.345 | 1.661 (0.576-4.792) |
|  |  |  |  | No | 2 | 6 | 29 |  |  |  |
|  |  |  |  | Survival |  |  |  |  |  |  |
|  |  |  |  | Yes | 2 | 6 | 28 | D | 0.422 | 0.649 (0.225-1.871) |
|  |  |  |  | No | 1 | 10 | 25 |  |  |  |
|  |  |  |  | GVHD3-4 |  |  |  |  |  |  |
|  |  |  |  | Yes | . | 5 | 21 | D | 0.300 | 0.544 (0.171-1.736) |
|  |  |  |  | No | 3 | 11 | 32 |  |  |  |
| rs107822 | RING1 | Chr 6: 33207798 | rs107822 | CMV | C/C | C/T | T/T |  |  |  |
|  | promoter |  |  | Yes | 3 | 18 | 11 | D | 0.675 | 1.241 (0.452-3.406) |
|  |  |  |  | No | 3 | 17 | 13 |  |  |  |
|  |  |  |  | Survival |  |  |  |  |  |  |
|  |  |  |  | Yes | 3 | 19 | 9 | D | 0.208 | 1.930 (0.689-5.402) |
|  |  |  |  | No | 3 | 16 | 15 |  |  |  |
|  |  |  |  | GVHD3-4 |  |  |  |  |  |  |
|  |  |  |  | Yes | 2 | 10 | 12 | D | 0.095 | 0.414 (0.145-1.177) |
|  |  |  |  | No | 4 | 25 | 12 |  |  |  |
| rs213210 | RING1 | Chr 6:33208047 | rs107822 | CMV | A/A | A/G | G/G |  |  |  |
|  | promoter |  |  | Yes | 7 | 15 | 10 | R | 0.294 | 2.030 (0.532-7.752) |
|  |  |  |  | No | 4 | 20 | 9 |  |  |  |
|  |  |  |  | Survival |  |  |  |  |  |  |
|  |  |  |  | Yes | 4 | 19 | 8 | R | 0.409 | 0.571 (0.150-2.181) |
|  |  |  |  | No | 7 | 16 | 11 |  |  |  |
| rs209132 | TRIM27 | Chr 6: 28899705 | rs209130 | CMV | A/A | A/G | G/G |  |  |  |
|  | ~3k downstream |  |  | Yes | 3 | 11 | 21 | D | 0.155 | 0.508 (0.199-1.298) |
|  |  |  |  | No | 4 | 17 | 16 |  |  |  |
|  |  |  |  | Survival |  |  |  |  |  |  |
|  |  |  |  | Yes | 3 | 16 | 17 | D | 0.479 | 1.397 (0.553-3.532) |
|  |  |  |  | No | 4 | 12 | 20 |  |  |  |
|  |  |  |  | GVHD3-4 |  |  |  |  |  |  |
|  |  |  |  | Yes | 1 | 9 | 16 | D | 0.195 | 0.525 (0.197-1.399) |
|  |  |  |  | No | 6 | 19 | 21 |  |  |  |
| rs209131 | TRIM27 | Chr 6: 28899978 | rs209130 | CMV | A/A | A/G | G/G |  |  |  |
|  | ~3k downstream |  |  | Yes | 3 | 19 | 13 | D | 0.675 | 0.812 (0.307-2.146) |
|  |  |  |  | No | 4 | 21 | 12 |  |  |  |
|  |  |  |  | Survival |  |  |  |  |  |  |
|  |  |  |  | Yes | 2 | 20 | 14 | R | 0.429 | 0.365 (0.066-2.018) |
|  |  |  |  | No | 5 | 20 | 11 |  |  |  |
|  |  |  |  | GVHD3-4 |  |  |  |  |  |  |
|  |  |  |  | Yes | . | 15 | 11 | D | 0.309 | 0.597 (0.220-1.622) |
|  |  |  |  | No | 7 | 25 | 14 |  |  |  |
| rs209130 | TRIM27 | Chr 6: 28900023 | rs209130 | CMV | C/C | C/T | T/T |  |  |  |
|  | ~3k downstream |  |  | Yes | 2 | 13 | 20 | D | 0.159 | 0.511 (0.200-1.306) |
|  |  |  |  | No | 3 | 19 | 15 |  |  |  |
|  |  |  |  | Survival |  |  |  |  |  |  |
|  |  |  |  | Yes | 3 | 16 | 17 | D | 0.814 | 1.118 (0.443-2.818) |
|  |  |  |  | No | 2 | 16 | 18 |  |  |  |
|  |  |  |  | GVHD3-4 |  |  |  |  |  |  |
|  |  |  |  | Yes | . | 11 | 15 | R | 0.152 | NA |
|  |  |  |  | No | 5 | 21 | 20 |  |  |  |
| rs1536215 | TRIM27 | Chr 6: 28900138 | rs209130 | CMV | C/C | C/G | G/G |  |  |  |
|  | ~3k downstream |  |  | Yes | 24 | 10 | 1 | R | 0.486 | NA |
|  |  |  |  | No | 28 | 9 | . |  |  |  |
|  |  |  |  | Survival |  |  |  |  |  |  |
|  |  |  |  | Yes | 28 | 8 | . | D | 0.293 | 0.571 (0.200-1.629) |
|  |  |  |  | No | 24 | 11 | 1 |  |  |  |
|  |  |  |  | GVHD3-4 |  |  |  |  |  |  |
|  |  |  |  | Yes | 18 | 8 | . | D | 0.670 | 1.259 (0.436-3.640) |
|  |  |  |  | No | 34 | 11 | 1 |  |  |  |
| rs139791445 | TRIM27 | Chr 6: 28900314 | rs209130 | CMV | C/C | C/G | G/G |  |  |  |
|  | ~3k downstream |  |  | Yes | 33 | 2 | . | D/A | 0.609 | 2.182 (0.189-25.1950) |
|  |  |  |  | No | 36 | 1 | . |  |  |  |
|  |  |  |  | Survival |  |  |  |  |  |  |
|  |  |  |  | Yes | 35 | 1 | . | D/A | 1.000 | 0.486 (0.042-5.608) |
|  |  |  |  | No | 34 | 2 | . |  |  |  |
| rs11244 | HLA-DOB | Chr 6: 32812947 | rs2071479 | CMV | A/A | A/G | G/G |  |  |  |
|  | 3’UTR |  |  | Yes | 1 | 11 | 22 | D | 0.649 | 0.800 (0.306-2.094) |
|  |  |  |  | No | 2 | 13 | 22 |  |  |  |
|  |  |  |  | Survival |  |  |  |  |  |  |
|  |  |  |  | Yes | 1 | 15 | 20 | D | 0.259 | 1.745 (0.661-4.606) |
|  |  |  |  | No | 2 | 9 | 24 |  |  |  |
|  |  |  |  | GVHD3-4 |  |  |  |  |  |  |
|  |  |  |  | Yes | 1 | 8 | 17 | D | 0.653 | 0.794 (0.291-2.168) |
|  |  |  |  | No | 2 | 16 | 27 |  |  |  |
| rs2070120 | HLA-DOB | Chr 6: 32813137 | rs2071479 | CMV | G/G | A/G | A/A |  |  |  |
|  | 3’UTR |  |  | Yes | 34 | 1 | . | D/A | 0.358 | 4.121 (0.437-38.830) |
|  |  |  |  | No | 33 | 4 | . |  |  |  |
|  |  |  |  | Survival |  |  |  |  |  |  |
|  |  |  |  | Yes | 35 | 1 | . | D/A | 0.357 | 4.375 (0.464-41.225) |
|  |  |  |  | No | 32 | 4 | . |  |  |  |
|  |  |  |  | GVHD3-4 |  |  |  |  |  |  |
|  |  |  |  | Yes | 25 | 1 | . | D/A | 0.647 | 2.381 (0.252-22.512) |
|  |  |  |  | No | 42 | 4 | . |  |  |  |
| rs56150445 | HLA-DOB | Chr 6: 32813158-  32813160 | rs2071479 | CMV | G/G | G/- | -/- |  |  |  |
|  | 3’UTR |  |  | Yes | 35 | . | . | NA | NA | NA |
|  |  |  |  | No | 37 | . | . |  |  |  |
|  |  |  |  | Survival |  |  |  |  |  |  |
|  |  |  |  | Yes | 36 | . | . | NA | NA | NA |
|  |  |  |  | No | 36 | . | . |  |  |  |
|  |  |  |  | GVHD3-4 |  |  |  |  |  |  |
|  |  |  |  | Yes | 26 | . | . | NA | NA | NA |
|  |  |  |  | No | 46 | . | . |  |  |  |
| rs41258084 | HLA-DOB | Chr 6: 32813180 | rs2071479 | CMV | C/C | C/T | T/T |  |  |  |
|  | 3’UTR |  |  | Yes | 29 | 6 | . | D/A | 0.509 | 1.707 (0.438-6.650) |
|  |  |  |  | No | 33 | 4 | . |  |  |  |
|  |  |  |  | Survival |  |  |  |  |  |  |
|  |  |  |  | Yes | 30 | 6 | . | D/A | 0.496 | 1.600 (0.411-6.232) |
|  |  |  |  | No | 32 | 4 | . |  |  |  |
|  |  |  |  | GVHD3-4 |  |  |  |  |  |  |
|  |  |  |  | Yes | 22 | 4 | . | D/A | 1.000 | 1.212 (0.309-4.760) |
|  |  |  |  | No | 40 | 6 | . |  |  |  |
| rs17220087 | HLA-DOB | Chr 6: 32813299 | rs2071479 | CMV | C/C | A/C | A/A |  |  |  |
|  | intron |  |  | Yes | 34 | 1 | . | D/A | 0.358 | 4.121 (0.437-38.830) |
|  |  |  |  | No | 33 | 4 | . |  |  |  |
|  |  |  |  | Survival |  |  |  |  |  |  |
|  |  |  |  | Yes | 35 | 1 | . | D/A | 0.357 | 4.375 (0.464-41.225) |
|  |  |  |  | No | 32 | 4 | . |  |  |  |
|  |  |  |  | GVHD3-4 |  |  |  |  |  |  |
|  |  |  |  | Yes | 25 | 1 | . | D/A | 0.647 | 2.381 (0.252-22.512) |
|  |  |  |  | No | 42 | 4 | . |  |  |  |
| rs2071479 | HLA-DOB | Chr 6: 32813335 | rs2071479 | CMV | C/C | C/T | T/T |  |  |  |
|  | intron |  |  | Yes | 35 | . | . | D/A | 0.493 | NA |
|  |  |  |  | No | 35 | 2 | . |  |  |  |
|  |  |  |  | Survival |  |  |  |  |  |  |
|  |  |  |  | Yes | 36 | . | . | D/A | 0.493 | NA |
|  |  |  |  | No | 34 | 2 | . |  |  |  |
|  |  |  |  | GVHD3-4 |  |  |  |  |  |  |
|  |  |  |  | Yes | 25 | 1 | . | D/A | 1.000 | 1.800 (0.108-30.036) |
|  |  |  |  | No | 45 | 1 | . |  |  |  |
| rs17213693 | HLA-DOB | Chr 6: 32813344 | rs2071479 | CMV | G/G | C/G | C/C |  |  |  |
|  | intron |  |  | Yes | 34 | 1 | . | D/A | 0.358 | 4.121 (0.437-8.830) |
|  |  |  |  | No | 33 | 4 | . |  |  |  |
|  |  |  |  | Survival |  |  |  |  |  |  |
|  |  |  |  | Yes | 35 | 1 | . | D/A | 0.357 | 4.375 (0.464-41.225) |
|  |  |  |  | No | 32 | 4 | . |  |  |  |
|  |  |  |  | GVHD3-4 |  |  |  |  |  |  |
|  |  |  |  | Yes | 25 | 1 | . | D/A | 0.647 | 2.3819 (0.252-22.512) |
|  |  |  |  | NO | 42 | 4 | . |  |  |  |
| AML recipient | | | | | | | | | | |
| rs9276982 | HLA-DOA  promoter | Chr 6: 33010438 | rs9276982 | CMV | A/A | A/G | G/G |  |  |  |
|  |  |  |  | Yes | 1 | 21 | 41 | D | 0.862 | 0.930 (0.410-2.111) |
|  |  |  |  | No | 1 | 14 | 26 |  |  |  |
|  |  |  |  | Survival |  |  |  |  |  |  |
|  |  |  |  | Yes | 2 | 20 | 33 | D | 0.318 | 1.511 (0.671-3.405) |
|  |  |  |  | No | . | 15 | 34 |  |  |  |
|  |  |  |  | GVHD3-4 |  |  |  |  |  |  |
|  |  |  |  | Yes | . | 6 | 16 | D | 0.360 | 0.617 (0.218-1.744) |
|  |  |  |  | No | 2 | 29 | 51 |  |  |  |
| rs71565361 | HLA-DOA | Chr 6: 33010551 | rs9276982 | CMV | A/A | A/C | C/C |  |  |  |
|  | promoter |  |  | Yes | . | . | 63 | NA | NA | NA |
|  |  |  |  | No | . | . | 41 |  |  |  |
|  |  |  |  | Survival |  |  |  |  |  |  |
|  |  |  |  | Yes | . | . | 55 | NA | NA | NA |
|  |  |  |  | No | . | . | 49 |  |  |  |
|  |  |  |  | GVHD3-4 |  |  |  |  |  |  |
|  |  |  |  | Yes | . | . | 22 | NA | NA | NA |
|  |  |  |  | No | . | . | 82 |  |  |  |
| rs79327197 | HLA-DOA | Chr 6: 33010635 | rs9276982 | CMV | A/A | A/G | G/G |  |  |  |
|  | promoter |  |  | Yes | 50 | 13 | . | D/A | 0.143 | 2.405 (0.725-7.973) |
|  |  |  |  | No | 37 | 4 | . |  |  |  |
|  |  |  |  | GVHD3-4 |  |  |  |  |  |  |
|  |  |  |  | Yes | 20 | 2 | . | D/A | 0.516 | 0.447 (0.094-2.121) |
|  |  |  |  | No | 67 | 15 | . |  |  |  |
| rs151190962 | HLA-DOA | Chr 6: 33010881 | rs9276982 | CMV | A/A | -/A | -/- |  |  |  |
|  | promoter |  |  | Yes | 60 | 3 | . | D/A | 1.000 | 0.950 (0.152-5.950) |
|  |  |  |  | No | 38 | 2 | . |  |  |  |
|  |  |  |  | Survival |  |  |  |  |  |  |
|  |  |  |  | Yes | 51 | 3 | . | D/A | 1.000 | 1.382 (0.221-8.640) |
|  |  |  |  | No | 47 | 2 | . |  |  |  |
|  |  |  |  | GVHD3-4 |  |  |  |  |  |  |
|  |  |  |  | Yes | 20 | 1 | . | D/A | 1.000 | 0.975 (0.103-9.211) |
|  |  |  |  | No | 78 | 4 | . |  |  |  |
| rs9282369 | HLA-DOA | Chr 6: 33011011-  33011018 | rs9276982 | CMV | -/- | -/T | T/T |  |  |  |
|  | promoter |  |  | Yes | 12 | 29 | 22 | R | 0.799 | 1.143 (0.409-3.195) |
|  |  |  |  | No | 7 | 19 | 15 |  |  |  |
|  |  |  |  | Survival |  |  |  |  |  |  |
|  |  |  |  | Yes | 10 | 26 | 19 | D | 0.816 | 1.100 (0.492-2.458) |
|  |  |  |  | No | 9 | 22 | 18 |  |  |  |
|  |  |  |  | GVHD3-4 |  |  |  |  |  |  |
|  |  |  |  | Yes | 5 | 10 | 7 | R | 0.544 | 1.429 (0.452-4.517) |
|  |  |  |  | No | 14 | 38 | 30 |  |  |  |
| rs2009658 | LTA | Chr 6: 31570467 | rs915654 | CMV | C/C | C/G | G/G |  |  |  |
|  | intron |  |  | Yes | 46 | 15 | 1 | D | 0.298 | 1.689 (0.626-4.559) |
|  |  |  |  | No | 34 | 6 | 1 |  |  |  |
|  |  |  |  | Survival |  |  |  |  |  |  |
|  |  |  |  | Yes | 40 | 15 | . | D | 0.197 | 0.533 (0.203-1.398) |
|  |  |  |  | No | 40 | 6 | 2 |  |  |  |
|  |  |  |  | GVHD3-4 |  |  |  |  |  |  |
|  |  |  |  | Yes | 17 | 4 | 1 | R | 0.383 | 3.810 (0.229-63.476) |
|  |  |  |  | No | 63 | 17 | 1 |  |  |  |
| rs111250247 | LTA | Chr 6: 31570719 | rs915654 | CMV | T/T | T/A | A/A |  |  |  |
|  | intron |  |  | Yes | 61 | 1 | . | D/A | 1.000 | 1.525 (0.093-25.087) |
|  |  |  |  | No | 40 | 1 | . |  |  |  |
|  |  |  |  | Survival |  |  |  |  |  |  |
|  |  |  |  | Yes | 53 | 2 | . | D/A | 0.497 | NA |
|  |  |  |  | No | 48 | . | . |  |  |  |
|  |  |  |  | GVHD3-4 |  |  |  |  |  |  |
|  |  |  |  | Yes | 22 | . | . | D/A | 1.000 | NA |
|  |  |  |  | No | 79 | 2 | . |  |  |  |
| rs915654 | LTA | Chr 6: 31570720 | rs915654 | CMV | A/A | A/T | T/T |  |  |  |
|  | intron |  |  | Yes | 18 | 33 | 11 | D | 0.271 | 1.700 (0.658-4.394) |
|  |  |  |  | No | 11 | 19 | 11 |  |  |  |
|  |  |  |  | Survival |  |  |  |  |  |  |
|  |  |  |  | Yes | 13 | 30 | 12 | R | 0.275 | 1.615 (0.681-3.834) |
|  |  |  |  | No | 16 | 22 | 10 |  |  |  |
|  |  |  |  | GVHD3-4 |  |  |  |  |  |  |
|  |  |  |  | Yes | 9 | 8 | 5 | R | 0.903 | 1.060 (0.412-2.731) |
|  |  |  |  | No | 20 | 44 | 17 |  |  |  |
| rs34324979 | HSPA1L | Chr 6: 31809994-  31809997 | rs2075800 | CMV | -/- | -/G | G/G |  |  |  |
|  | 3’UTR |  |  | Yes | 5 | 13 | 45 | R | 0.154 | NA |
|  |  |  |  | No | . | 12 | 29 |  |  |  |
|  |  |  |  | Survival |  |  |  |  |  |  |
|  |  |  |  | Yes | 3 | 15 | 37 | D | 0.355 | 1.500 (0.634-3.548) |
|  |  |  |  | No | 2 | 10 | 37 |  |  |  |
|  |  |  |  | GVHD3-4 | -/- | -/G | G/G |  |  |  |
|  |  |  |  | Yes | 1 | 9 | 12 | D | 0.053 | 2.583 (0.971-6.876) |
|  |  |  |  | NO | 4 | 16 | 62 |  |  |  |
| rs2075800 | HSPA1L | Chr 6: 31810169 | rs2075800 | CMV | C/C | C/T | T/T |  |  |  |
|  | exon |  |  | Yes | 27 | 28 | 7 | R | 0.080 | 0.395 (0.136-1.140) |
|  |  |  |  | No | 15 | 16 | 10 |  |  |  |
|  |  |  |  | Survival |  |  |  |  |  |  |
|  |  |  |  | Yes | 19 | 24 | 11 | D | 0.225 | 0.614 (0.278-1.354) |
|  |  |  |  | No | 23 | 20 | 6 |  |  |  |
|  |  |  |  | GVHD3-4 |  |  |  |  |  |  |
|  |  |  |  | Yes | 9 | 11 | 2 | R | 0.517 | 0.440 (0.093-2.090) |
|  |  |  |  | No | 33 | 33 | 15 |  |  |  |
| rs2227956 | HSPA1L | Chr 6: 31810495 | rs2075800 | CMV | A/A | A/G | G/G |  |  |  |
|  | exon |  |  | Yes | 48 | 11 | 3 | D | 0.303 | 0.628 (0.259-1.525) |
|  |  |  |  | No | 28 | 12 | 1 |  |  |  |
|  |  |  |  | Survival |  |  |  |  |  |  |
|  |  |  |  | Yes | 40 | 12 | 2 | D | 0.944 | 1.032 (0.428-1.485) |
|  |  |  |  | No | 36 | 11 | 2 |  |  |  |
|  |  |  |  | GVHD3-4 |  |  |  |  |  |  |
|  |  |  |  | Yes | 15 | 7 | . | D | 0.500 | 1.423 (0.508-3.985) |
|  |  |  |  | No | 61 | 16 | 4 |  |  |  |
| rs3130048 | BAG6 | Chr 6: 31645962 | rs2242656 | CMV | C/C | C/T | T/T |  |  |  |
|  | intron |  |  | Yes | 6 | 23 | 33 | R | 0.472 | 2.089 (0.401-10.897) |
|  |  |  |  | No | 2 | 117 | 22 |  |  |  |
|  |  |  |  | Survival |  |  |  |  |  |  |
|  |  |  |  | Yes | 4 | 19 | 32 | D | 0.298 | 0.661 (0.303-1.442) |
|  |  |  |  | No | 4 | 21 | 23 |  |  |  |
|  |  |  |  | GVHD3-4 |  |  |  |  |  |  |
|  |  |  |  | Yes | 1 | 8 | 13 | D | 0.546 | 0.746 (0.287-1.938) |
|  |  |  |  | No | 7 | 32 | 42 |  |  |  |
| rs2844464 | BAG6 | Chr 6: 31646214 | rs2242656 | CMV | A/A | A/G | G/G |  |  |  |
|  | intron |  |  | Yes | 5 | 16 | 41 | R | 0.398 | 3.509 (0.395-31.188) |
|  |  |  |  | No | 1 | 16 | 24 |  |  |  |
|  |  |  |  | Survival |  |  |  |  |  |  |
|  |  |  |  | Yes | 3 | 19 | 33 | D | 0.484 | 1.333 (0.595-2.988) |
|  |  |  |  | No | 3 | 13 | 32 |  |  |  |
|  |  |  |  | GVHD3-4 |  |  |  |  |  |  |
|  |  |  |  | Yes | 2 | 9 | 11 | D | 0.151 | 2.000 (0.770-5.197) |
|  |  |  |  | No | 4 | 23 | 54 |  |  |  |
| rs2242656 | BAG6 | Chr 6: 31646325 | rs2242656 | CMV | C/C | C/T | T/T |  |  |  |
|  | intron |  |  | Yes | 5 | 16 | 41 | R | 0.398 | 3.509 (0.395-31.188) |
|  |  |  |  | No | 1 | 16 | 24 |  |  |  |
|  |  |  |  | Survival |  |  |  |  |  |  |
|  |  |  |  | Yes | 3 | 19 | 33 | D | 0.484 | 1.333 (0.595-2.988) |
|  |  |  |  | No | 3 | 13 | 32 |  |  |  |
|  |  |  |  | GVHD3-4 |  |  |  |  |  |  |
|  |  |  |  | Yes | 2 | 9 | 11 | D | 0.151 | 2.000 (0.770-5.197) |
|  |  |  |  | No | 4 | 23 | 54 |  |  |  |
| rs107822 | RING1 | Chr 6: 33207798 | rs107822 | CMV | C/C | C/T | T/T |  |  |  |
|  | promoter |  |  | Yes | 8 | 29 | 25 | R | 0.759 | 1.370 (0.384-4.885) |
|  |  |  |  | No | 4 | 21 | 16 |  |  |  |
|  |  |  |  | Survival |  |  |  |  |  |  |
|  |  |  |  | Yes | 8 | 24 | 23 | R | 0.327 | 1.872 (0.526-6.658) |
|  |  |  |  | No | 4 | 26 | 18 |  |  |  |
|  |  |  |  | GVHD3-4 |  |  |  |  |  |  |
|  |  |  |  | Yes | 1 | 11 | 10 | R | 0.454 | 0.303 (0.037-2.486) |
|  |  |  |  | No | 11 | 39 | 31 |  |  |  |
| rs213210 | RING1 | Chr 6:33208047 | rs107822 | CMV | A/A | A/G | G/G |  |  |  |
|  | promoter |  |  | Yes | 15 | 30 | 17 | D | 0.639 | 1.229 (0.195-2.912) |
|  |  |  |  | No | 11 | 17 | 13 |  |  |  |
|  |  |  |  | Survival |  |  |  |  |  |  |
|  |  |  |  | Yes | 14 | 24 | 17 | D | 0.670 | 0.830 (0.353-1.954) |
|  |  |  |  | No | 12 | 23 | 13 |  |  |  |
|  |  |  |  | GVHD3-4 |  |  |  |  |  |  |
|  |  |  |  | Yes | 5 | 11 | 6 | R | 0.759 | 0.840 (0.276-2.560) |
|  |  |  |  | No | 21 | 36 | 24 |  |  |  |
| rs209132 | TRIM27 | Chr 6: 28899705 | rs209130 | Survival | A/A | A/G | G/G |  |  |  |
|  | ~3k downstream |  |  | Yes | 5 | 25 | 25 | D | 0.274 | 1.543 (0.708-3.362) |
|  |  |  |  | No | 2 | 19 | 27 |  |  |  |
|  |  |  |  | GVHD3-4 |  |  |  |  |  |  |
|  |  |  |  | Yes | . | 7 | 15 | D | 0.061 | 0.392 (0.145-1.065) |
|  |  |  |  | No | 7 | 37 | 37 |  |  |  |
| rs209131 | TRIM27 | Chr 6: 28899978 | rs209130 | Survival | A/A | A/G | G/G |  |  |  |
|  | ~3k downstream |  |  | Yes | 11 | 33 | 11 | D | 0.079 | 2.194 (0.904-5.325) |
|  |  |  |  | No | 7 | 24 | 17 |  |  |  |
|  |  |  |  | GVHD3-4 |  |  |  |  |  |  |
|  |  |  |  | Yes | 3 | 11 | 8 | D | 0.275 | 0.574 (0.210-1.567) |
|  |  |  |  | No | 15 | 46 | 20 |  |  |  |
| rs209130 | TRIM27 | Chr 6: 28900023 | rs209130 | CMV | C/C | C/T | T/T |  |  |  |
|  | ~3k downstream |  |  | Yes | 2 | 29 | 31 | D | 0.066 | 0.464 (0.203-1.059) |
|  |  |  |  | No | 5 | 23 | 13 |  |  |  |
|  |  |  |  | Survival |  |  |  |  |  |  |
|  |  |  |  | Yes | 5 | 29 | 21 | D | 0.319 | 1.490 (0.679-3.266) |
|  |  |  |  | No | 2 | 23 | 23 |  |  |  |
|  |  |  |  | GVHD3-4 |  |  |  |  |  |  |
|  |  |  |  | Yes | . | 9 | 13 | D | 0.080 | 0.429 (0.164-1.122) |
|  |  |  |  | No | 7 | 43 | 31 |  |  |  |
| rs1536215 | TRIM27 | Chr 6: 28900138 | rs209130 | CMV | C/C | C/G | G/G |  |  |  |
|  | ~3k downstream |  |  | Yes | 45 | 17 | . | D/A | 0.947 | 1.030 (0.424-2.504) |
|  |  |  |  | No | 30 | 11 | . |  |  |  |
|  |  |  |  | Survival |  |  |  |  |  |  |
|  |  |  |  | Yes | 38 | 17 | . | D/A | 0.363 | 1.505 (0.622-3.640) |
|  |  |  |  | No | 37 | 11 | . |  |  |  |
|  |  |  |  | GVHD3-4 |  |  |  |  |  |  |
|  |  |  |  | Yes | 14 | 8 | . | D/A | 0.275 | 1.743 (0.638-4.760) |
|  |  |  |  | No | 61 | 20 | . |  |  |  |
| rs139791445 | TRIM27 | Chr 6: 28900314 | rs209130 | CMV | C/C | C/G | G/G |  |  |  |
|  | ~3k downstream |  |  | Yes | 59 | 3 | . | D/A | 0.278 | NA |
|  |  |  |  | No | 40 | . | . |  |  |  |
|  |  |  |  | Survival |  |  |  |  |  |  |
|  |  |  |  | Yes | 52 | 2 | . | D/A | 1.000 | 1.808 (0.159-20.588) |
|  |  |  |  | No | 47 | 1 | . |  |  |  |
|  |  |  |  | GVHD3-4 |  |  |  |  |  |  |
|  |  |  |  | Yes | 21 | 1 | . | D/A | 0.521 | 1.857 (0.161-21.486) |
|  |  |  |  | No | 78 | 2 | . |  |  |  |
| rs11244 | HLA-DOB | Chr 6: 32812947 | rs2071479 | CMV | A/A | A/G | G/G |  |  |  |
|  | 3’UTR |  |  | Yes | 3 | 21 | 39 | D | 0.071 | 0.482 (0.217-1.071) |
|  |  |  |  | No | 5 | 18 | 18 |  |  |  |
|  |  |  |  | Survival |  |  |  |  |  |  |
|  |  |  |  | Yes | 6 | 21 | 28 | R | 0.276 | 2.878 (0.553-14.977) |
|  |  |  |  | No | 2 | 18 | 29 |  |  |  |
|  |  |  |  | GVHD3-4 |  |  |  |  |  |  |
|  |  |  |  | Yes | 3 | 9 | 10 | D | 0.321 | 1.611 (0.625-4.152) |
|  |  |  |  | No | 5 | 30 | 47 |  |  |  |
| rs2070120 | HLA-DOB  3’UTR | Chr 6: 32813137 | rs2071479 | Survival | A/A | A/G | G/G |  |  |  |
|  |  |  |  | Yes | . | 8 | 47 | D/A | 0.309 | 0.522 (0.147-1.856) |
|  |  |  |  | No | . | 4 | 45 |  |  |  |
|  |  |  |  | GVHD3-4 |  |  |  |  |  |  |
|  |  |  |  | Yes | . | 2 | 20 | D/A | 1.000 | 1.389 (0.281-6.858) |
|  |  |  |  | No | . | 10 | 72 |  |  |  |
| rs56150445 | HLA-DOB | Chr 6: 32813158-  32813160 | rs2071479 | CMV | -/- | -/G | G/G |  |  |  |
|  | 3’UTR |  |  | Yes | . | . | 63 | D/A | 0.153 | NA |
|  |  |  |  | No | . | 2 | 39 |  |  |  |
|  |  |  |  | Survival |  |  |  |  |  |  |
|  |  |  |  | Yes | . | 1 | 54 | D/A | 1.000 | 1.125 (0.068-18.481) |
|  |  |  |  | No | . | 1 | 48 |  |  |  |
|  |  |  |  | GVHD3-4 |  |  |  |  |  |  |
|  |  |  |  | Yes | . | . | 22 | D/A | 1.000 | NA |
|  |  |  |  | No | . | 2 | 80 |  |  |  |
| rs41258084 | HLA-DOB | Chr 6: 32813180 | rs2071479 | CMV | C/C | C/T | T/T |  |  |  |
|  | 3’UTR |  |  | Yes | 56 | 6 | 1 | D | 0.562 | 0.708 (0.220-2.284) |
|  |  |  |  | No | 34 | 6 | . |  |  |  |
|  |  |  |  | Survival |  |  |  |  |  |  |
|  |  |  |  | Yes | 49 | 5 | . | D | 0.281 | 0.523 (0.159-1.72) |
|  |  |  |  | No | 41 | 7 | 1 |  |  |  |
|  |  |  |  | GVHD3-4 |  |  |  |  |  |  |
|  |  |  |  | Yes | 18 | 3 | 1 | R | 0.214 | NA |
|  |  |  |  | No | 72 | 9 | 0 |  |  |  |
| rs17220087 | HLA-DOB | Chr 6: 32813299 | rs2071479 | CMV | A/A | A/C | C/C |  |  |  |
|  | intron |  |  | Yes | . | 10 | 53 | D/A | 0.121 | 0.279 (0.058-1.347) |
|  |  |  |  | No | . | 2 | 38 |  |  |  |
|  |  |  |  | Survival |  |  |  |  |  |  |
|  |  |  |  | Yes | . | 7 | 47 | D/A | 0.663 | 0.763 (0.225-2.582) |
|  |  |  |  | No | . | 5 | 44 |  |  |  |
|  |  |  |  | GVHD3-4 |  |  |  |  |  |  |
|  |  |  |  | Yes | . | 2 | 20 | D/A | 1.000 | 1.408 (0.285-6.957) |
|  |  |  |  | No | . | 10 | 71 |  |  |  |
| rs2071479 | HLA-DOB | Chr 6: 32813335 | rs2071479 | CMV | C/C | C/T | T/T |  |  |  |
|  | intron |  |  | Yes | 61 | 1 | 1 | D | 0.053 | 0.186 (0.036-0.972) |
|  |  |  |  | No | 34 | 5 | 1 |  |  |  |
|  |  |  |  | Survival |  |  |  |  |  |  |
|  |  |  |  | Yes | 49 | 4 | 1 | D | 0.718 | 1.565 (0.354-6.920) |
|  |  |  |  | No | 46 | 2 | 1 |  |  |  |
|  |  |  |  | GVHD3-4 |  |  |  |  |  |  |
|  |  |  |  | Yes | 19 | 3 | . | D | 0.363 | 2.400 (0.526-10.941) |
|  |  |  |  | No | 76 | 3 | 2 |  |  |  |
| rs17213693 | HLA-DOB | Chr 6: 32813344 | rs2071479 | Survival | C/C | C/G | G/G |  |  |  |
|  | intron |  |  | Yes | 1 | 8 | 45 | D | 0.339 | 1.760 (0.547-5.668) |
|  |  |  |  | No | . | 5 | 44 |  |  |  |
|  |  |  |  | GVHD3-4 |  |  |  |  |  |  |
|  |  |  |  | Yes | . | 2 | 20 | D | 0.729 | 0.575 (0.119-2.785) |
|  |  |  |  | NO | 1 | 11 | 69 |  |  |  |

D: dominant model (AA vs. Aa + aa); R: recessive model (AA + Aa vs. aa); A: additive model (AA vs. Aa vs. aa), in which “A” was defined as a higher frequency allele and the lower was “a”.

Table S3. The complete data for genetic analysis of SNPs in donor-recipient pairs group, except for the BMT-related SNPs.

| **SNP** | **Gene** | **Chromosome position (bp)** | **Source** | **Outcome /status** | **Genotypes between the Donor-Recipient Pairs** | | **Test** | ***p*-value** | **OR (95% CI)** |
| --- | --- | --- | --- | --- | --- | --- | --- | --- | --- |
| rs3130048 | BAG6 | Chr 6: 31645962 | rs2242656 | CMV | Matched | Unmatched |  |  |  |
|  | intron |  |  | Yes | 30 | 5 | Chi-square | 0.419 | 1.655 (0.485-5.654) |
|  |  |  |  | No | 29 | 8 |  |  |  |
|  |  |  |  | Survival |  |  |  |  |  |
|  |  |  |  | Yes | 29 | 7 | Chi-square | 0.759 | 0.829 (0.249-2.762) |
|  |  |  |  | No | 30 | 6 |  |  |  |
|  |  |  |  | GVHD3-4 |  |  |  |  |  |
|  |  |  |  | Yes | 20 | 6 | Fisher's exact test | 0.526 | 0.598 (0.177-2.019) |
|  |  |  |  | No | 39 | 7 |  |  |  |
| rs2844464 | BAG6 | Chr 6: 31646214 | rs2242656 | CMV | Matched | Unmatched |  |  |  |
|  | intron |  |  | Yes | 29 | 6 | Fisher's exact test | 0.146 | 0.276 (0.052-1.473) |
|  |  |  |  | No | 35 | 2 |  |  |  |
|  |  |  |  | Survival |  |  |  |  |  |
|  |  |  |  | Yes | 34 | 2 | Fisher's exact test | 0.260 | 3.400 (0.638-18.132) |
|  |  |  |  | No | 30 | 6 |  |  |  |
|  |  |  |  | GVHD3-4 |  |  |  |  |  |
|  |  |  |  | Yes | 23 | 3 | Fisher's exact test | 1.000 | 0.935 (0.205-4.274) |
|  |  |  |  | No | 41 | 5 |  |  |  |
| rs2242656 | BAG6 | Chr 6: 31646325 | rs2242656 | CMV | Matched | Unmatched |  |  |  |
|  | intron |  |  | Yes | 31 | 4 | Fisher's exact test | 0.193 | 0.215 (0.023-2.029) |
|  |  |  |  | No | 36 | 1 |  |  |  |
|  |  |  |  | Survival |  |  |  |  |  |
|  |  |  |  | Yes | 34 | 2 | Fisher's exact test | 1.000 | 1.545 (0.242-9.850) |
|  |  |  |  | No | 33 | 3 |  |  |  |
|  |  |  |  | GVHD3-4 |  |  |  |  |  |
|  |  |  |  | Yes | 25 | 1 | Fisher's exact test | 0.647 | 2.381 (0.252-22.512) |
|  |  |  |  | No | 42 | 4 |  |  |  |
| rs107822 | RING1 | Chr 6: 33207798 | rs107822 | CMV | Matched | Unmatched |  |  |  |
|  | promoter |  |  | Yes | 23 | 8 | Chi-square | 1.000 | 1.000 (0.321-3.120) |
|  |  |  |  | No | 23 | 8 |  |  |  |
|  |  |  |  | Survival |  |  |  |  |  |
|  |  |  |  | Yes | 23 | 6 | Chi-square | 0.388 | 1.667 (0.520-5.346) |
|  |  |  |  | No | 23 | 10 |  |  |  |
|  |  |  |  | GVHD3-4 |  |  |  |  |  |
|  |  |  |  | Yes | 16 | 8 | Chi-square | 0.282 | 0.533 (0.168-1.689) |
|  |  |  |  | No | 30 | 8 |  |  |  |
| rs213210 | RING1 | Chr 6:33208047 | rs107822 | CMV | Matched | Unmatched |  |  |  |
|  | promoter |  |  | Yes | 23 | 8 | Chi-square | 0.409 | 1.581 (0.532-4.704) |
|  |  |  |  | No | 20 | 11 |  |  |  |
|  |  |  |  | Survival |  |  |  |  |  |
|  |  |  |  | Yes | 21 | 8 | Chi-square | 0.624 | 1.313 (0.442-3.902) |
|  |  |  |  | No | 22 | 11 |  |  |  |
|  |  |  |  | GVHD3-4 |  |  |  |  |  |
|  |  |  |  | Yes | 15 | 9 | Chi-square | 0.352 | 0.595 (0.199-1.784) |
|  |  |  |  | No | 28 | 10 |  |  |  |
| rs209132 | TRIM27 | Chr 6: 28899705 | rs209130 | CMV | Matched | Unmatched |  |  |  |
|  | ~3k downstream |  |  | Yes | 28 | 7 | Chi-square | 0.307 | 1.760 (0.592-5.237) |
|  |  |  |  | No | 25 | 11 |  |  |  |
|  |  |  |  | Survival |  |  |  |  |  |
|  |  |  |  | Yes | 27 | 9 | Chi-square | 0.945 | 1.038 (0.356-3.026) |
|  |  |  |  | No | 26 | 9 |  |  |  |
|  |  |  |  | GVHD3-4 |  |  |  |  |  |
|  |  |  |  | Yes | 18 | 8 | Chi-square | 0.425 | 0.643 (0.216-1.912) |
|  |  |  |  | No | 35 | 10 |  |  |  |
| rs209131 | TRIM27 | Chr 6: 28899978 | rs209130 | CMV | Matched | Unmatched |  |  |  |
|  | ~3k downstream |  |  | Yes | 23 | 12 | Chi-square | 0.872 | 1.083 (0.409-2.871) |
|  |  |  |  | No | 23 | 13 |  |  |  |
|  |  |  |  | Survival |  |  |  |  |  |
|  |  |  |  | Yes | 23 | 13 | Chi-square | 0.872 | 0.923 (0.348-2.446) |
|  |  |  |  | No | 23 | 12 |  |  |  |
|  |  |  |  | GVHD3-4 |  |  |  |  |  |
|  |  |  |  | Yes | 15 | 11 | Chi-square | 0.341 | 0.616 (0.226-1.677) |
|  |  |  |  | No | 31 | 14 |  |  |  |
| rs209130 | TRIM27 | Chr 6: 28900023 | rs209130 | CMV | Matched | Unmatched |  |  |  |
|  | ~3k downstream |  |  | Yes | 25 | 10 | Chi-square | 0.664 | 1.250 (0.456-3.428) |
|  |  |  |  | No | 24 | 12 |  |  |  |
|  |  |  |  | Survival |  |  |  |  |  |
|  |  |  |  | Yes | 24 | 12 | Chi-square | 0.664 | 0.800 (0.292-2.194) |
|  |  |  |  | No | 25 | 10 |  |  |  |
| rs1536215 | TRIM27 | Chr 6: 28900138 | rs209130 | CMV | Matched | Unmatched |  |  |  |
|  | ~3k downstream |  |  | Yes | 27 | 8 | Chi-square | 0.949 | 0.964 (0.317-2.936) |
|  |  |  |  | No | 28 | 8 |  |  |  |
|  |  |  |  | Survival |  |  |  |  |  |
|  |  |  |  | Yes | 30 | 6 | Chi-square | 0.230 | 2.000 (0.638-6.271) |
|  |  |  |  | No | 25 | 10 |  |  |  |
|  |  |  |  | GVHD3-4 |  |  |  |  |  |
|  |  |  |  | Yes | 20 | 6 | Chi-square | 0.934 | 0.952 (0.301-3.013) |
|  |  |  |  | No | 35 | 10 |  |  |  |
| rs139791445 | TRIM27 | Chr 6: 28900314 | rs209130 | CMV | Matched | Unmatched |  |  |  |
|  | ~3k downstream |  |  | Yes | 33 | 2 | Fisher's exact test | 1.000 | 1.500 (0.235-9.569) |
|  |  |  |  | No | 33 | 3 |  |  |  |
|  |  |  |  | Survival |  |  |  |  |  |
|  |  |  |  | Yes | 35 | 1 | Fisher's exact test | 0.199 | 4.516 (0.479-42.591) |
|  |  |  |  | No | 31 | 4 |  |  |  |
|  |  |  |  | GVHD3-4 |  |  |  |  |  |
|  |  |  |  | Yes | 24 | 2 | Fisher's exact test | 1.000 | 0.857 (0.134-5.496) |
|  |  |  |  | No | 42 | 3 |  |  |  |
| rs11244 | HLA-DOB | Chr 6: 32812947 | rs2071479 | CMV | Matched | Unmatched |  |  |  |
|  | 3’UTR |  |  | Yes | 29 | 5 | Chi-square | 0.204 | 2.148 (0.650-7.94) |
|  |  |  |  | No | 27 | 10 |  |  |  |
|  |  |  |  | Survival |  |  |  |  |  |
|  |  |  |  | Yes | 30 | 6 | Chi-square | 0.350 | 1.731 (0.543-5.515) |
|  |  |  |  | No | 26 | 9 |  |  |  |
|  |  |  |  | GVHD3-4 |  |  |  |  |  |
|  |  |  |  | Yes | 20 | 6 | Chi-square | 0.760 | 0.833 (0.259-2.681) |
|  |  |  |  | No | 36 | 9 |  |  |  |
| rs2070120 | HLA-DOB | Chr 6: 32813137 | rs2071479 | CMV | Matched | Unmatched |  |  |  |
|  | 3’UTR |  |  | Yes | 29 | 5 | Fisher's exact test | 0.729 | 0.703 (0.172-2.869) |
|  |  |  |  | No | 33 | 4 |  |  |  |
|  |  |  |  | Survival |  |  |  |  |  |
|  |  |  |  | Yes | 31 | 5 | Fisher's exact test | 1.000 | 0.800 (0.196-3.263) |
|  |  |  |  | No | 31 | 4 |  |  |  |
|  |  |  |  | GVHD3-4 |  |  |  |  |  |
|  |  |  |  | Yes | 24 | 2 | Fisher's exact test | 0.470 | 2.211 (0.423-11.539) |
|  |  |  |  | No | 38 | 7 |  |  |  |
| rs56150445 | HLA-DOB | Chr 6: 32813158-  32813160 | rs2071479 | CMV | Matched | Unmatched |  |  |  |
|  | 3’UTR |  |  | Yes | 34 | . | NA | NA | NA |
|  |  |  |  | No | 37 | . |  |  |  |
|  |  |  |  | Survival |  |  |  |  |  |
|  |  |  |  | Yes | 36 | . | NA | NA | NA |
|  |  |  |  | No | 35 | . |  |  |  |
|  |  |  |  | GVHD3-4 |  |  |  |  |  |
|  |  |  |  | Yes | 26 | . | NA | NA | NA |
|  |  |  |  | No | 45 | . |  |  |  |
| rs41258084 | HLA-DOB | Chr 6: 32813180 | rs2071479 | CMV | Matched | Unmatched |  |  |  |
|  | 3’UTR |  |  | Yes | 28 | 6 | Fisher's exact test | 0.141 | 0.267 (0.050-1.425) |
|  |  |  |  | No | 35 | 2 |  |  |  |
|  |  |  |  | Survival |  |  |  |  |  |
|  |  |  |  | Yes | 33 | 3 | Fisher's exact test | 0.478 | 1.833 (0.403-8.335) |
|  |  |  |  | No | 30 | 5 |  |  |  |
|  |  |  |  | GVHD3-4 |  |  |  |  |  |
|  |  |  |  | Yes | 22 | 4 | Fisher's exact test | 0.453 | 0.537 (0.122-2.356) |
|  |  |  |  | No | 41 | 4 |  |  |  |
| rs17220087 | HLA-DOB | Chr 6: 32813299 | rs2071479 | CMV | Matched | Unmatched |  |  |  |
|  | intron |  |  | Yes | 28 | 6 | Fisher's exact test | 0.504 | 0.566 (0.145-2.208) |
|  |  |  |  | No | 33 | 4 |  |  |  |
|  |  |  |  | Survival |  |  |  |  |  |
|  |  |  |  | Yes | 31 | 5 | Fisher's exact test | 1.000 | 1.033 (0.271-3.936) |
|  |  |  |  | No | 30 | 5 |  |  |  |
|  |  |  |  | GVHD3-4 |  |  |  |  |  |
|  |  |  |  | Yes | 24 | 2 | Fisher's exact test | 0.307 | 2.595 (0.507-13.275) |
|  |  |  |  | No | 37 | 8 |  |  |  |
| rs2071479 | HLA-DOB | Chr 6: 32813335 | rs2071479 | CMV | Matched | Unmatched |  |  |  |
|  | intron |  |  | Yes | 34 | . | Fisher's exact test | 1.000 | NA |
|  |  |  |  | No | 36 | 1 |  |  |  |
|  |  |  |  | Survival |  |  |  |  |  |
|  |  |  |  | Yes | 36 | . | Fisher's exact test | 0.493 | NA |
|  |  |  |  | No | 34 | 1 |  |  |  |
|  |  |  |  | GVHD3-4 |  |  |  |  |  |
|  |  |  |  | Yes | 26 | . | Fisher's exact test | 1.000 | NA |
|  |  |  |  | No | 44 | 1 |  |  |  |
| rs17213693 | HLA-DOB | Chr 6: 32813344 | rs2071479 | CMV | Matched | Unmatched |  |  |  |
|  | intron |  |  | Yes | 29 | 5 | Fisher's exact test | 0.729 | 0.703 (0.172-2.869) |
|  |  |  |  | No | 33 | 4 |  |  |  |
|  |  |  |  | Survival |  |  |  |  |  |
|  |  |  |  | Yes | 31 | 5 | Fisher's exact test | 1.000 | 0.800 (0.196-3.263) |
|  |  |  |  | No | 31 | 4 |  |  |  |
|  |  |  |  | GVHD3-4 |  |  |  |  |  |
|  |  |  |  | Yes | 24 | 2 | Fisher's exact test | 0.470 | 2.211 (0.423-11.539) |
|  |  |  |  | No | 38 | 7 |  |  |  |
| AML donor-recipient pairs | | | | | | | | | |
| rs3130048 | BAG6 | Chr 6: 31645962 | rs2242656 | CMV | Matched | Unmatched |  |  |  |
|  | intron |  |  | Yes | 54 | 8 | Chi-square | 0.226 | 1.898 (0.666-5.414) |
|  |  |  |  | No | 32 | 9 |  |  |  |
|  |  |  |  | Survival |  |  |  |  |  |
|  |  |  |  | Yes | 46 | 9 | Chi-square | 0.967 | 1.022 (0.360-2.899) |
|  |  |  |  | No | 40 | 9 |  |  |  |
| rs2844464 | BAG6 | Chr 6: 31646214 | rs2242656 | CMV | Matched | Unmatched |  |  |  |
|  | intron |  |  | Yes | 53 | 9 | Chi-square | 0.737 | 0.818 (0.253-2.641) |
|  |  |  |  | No | 35 | 5 |  |  |  |
|  |  |  |  | Survival |  |  |  |  |  |
|  |  |  |  | Yes | 49 | 6 | Chi-square | 0.395 | 1.633 (0.523-5.096) |
|  |  |  |  | No | 40 | 8 |  |  |  |
|  |  |  |  | GVHD3-4 |  |  |  |  |  |
|  |  |  |  | Yes | 17 | 5 | Fisher's exact test | 0.172 | 0.425 (0.126-1.431) |
|  |  |  |  | No | 72 | 9 |  |  |  |
| rs2242656 | BAG6 | Chr 6: 31646325 | rs2242656 | CMV | Matched | Unmatched |  |  |  |
|  | intron |  |  | Yes | 53 | 9 | Chi-square | 0.737 | 0.818 (0.253-2.641) |
|  |  |  |  | No | 36 | 5 |  |  |  |
|  |  |  |  | Survival |  |  |  |  |  |
|  |  |  |  | Yes | 49 | 6 | Chi-square | 0.395 | 1.633 (0.523-5.096) |
|  |  |  |  | No | 40 | 8 |  |  |  |
|  |  |  |  | GVHD3-4 |  |  |  |  |  |
|  |  |  |  | Yes | 17 | 5 | Fisher's exact test | 0.172 | 0.425 (0.126-1.431) |
|  |  |  |  | No | 72 | 9 |  |  |  |
| rs107822 | RING1 | Chr 6: 33207798 | rs107822 | CMV | Matched | Unmatched |  |  |  |
|  | promoter |  |  | Yes | 34 | 26 | Chi-square | 0.666 | 0.837 (0.373-1.879) |
|  |  |  |  | No | 25 | 16 |  |  |  |
|  |  |  |  | GVHD3-4 |  |  |  |  |  |
|  |  |  |  | Yes | 13 | 9 | Chi-square | 0.942 | 1.036 (0.397-2.707) |
|  |  |  |  | No | 46 | 33 |  |  |  |
| rs213210 | RING1 | Chr 6:33208047 | rs107822 | CMV | Matched | Unmatched |  |  |  |
|  | promoter |  |  | Yes | 34 | 26 | Chi-square | 0.354 | 0.678 (0.298-1.544) |
|  |  |  |  | No | 27 | 14 |  |  |  |
|  |  |  |  | Survival |  |  |  |  |  |
|  |  |  |  | Yes | 29 | 24 | Chi-square | 0.220 | 0.604 (0.269-1.355) |
|  |  |  |  | No | 32 | 16 |  |  |  |
|  |  |  |  | GVHD3-4 |  |  |  |  |  |
|  |  |  |  | Yes | 13 | 9 | Chi-square | 0.887 | 0.933 (0.356-2.442) |
|  |  |  |  | No | 48 | 31 |  |  |  |
| rs209132 | TRIM27 | Chr 6: 28899705 | rs209130 | CMV | Matched | Unmatched |  |  |  |
|  | ~3k downstream |  |  | Yes | 42 | 20 | Chi-square | 0.390 | 0.677 (0.278-1.649) |
|  |  |  |  | No | 31 | 10 |  |  |  |
|  |  |  |  | Survival |  |  |  |  |  |
|  |  |  |  | Yes | 39 | 16 | Chi-square | 0.993 | 1.004 (0.428-2.353) |
|  |  |  |  | No | 34 | 14 |  |  |  |
|  |  |  |  | GVHD3-4 |  |  |  |  |  |
|  |  |  |  | Yes | 15 | 7 | Chi-square | 0.754 | 0.850 (0.307-2.354) |
|  |  |  |  | No | 58 | 23 |  |  |  |
| rs209131 | TRIM27 | Chr 6: 28899978 | rs209130 | Survival | Matched | Unmatched |  |  |  |
|  | ~3k downstream |  |  | Yes | 37 | 18 | Chi-square | 0.469 | 1.347 (0.601-3.019) |
|  |  |  |  | No | 29 | 19 |  |  |  |
|  |  |  |  | GVHD3-4 |  |  |  |  |  |
|  |  |  |  | Yes | 12 | 10 | Chi-square | 0.293 | 0.600 (0.230-1.564) |
|  |  |  |  | No | 54 | 27 |  |  |  |
| rs209130 | TRIM27 | Chr 6: 28900023 | rs209130 | CMV | Matched | Unmatched |  |  |  |
|  | ~3k downstream |  |  | Yes | 41 | 21 | Chi-square | 0.624 | 0.837 (0.808-1.898) |
|  |  |  |  | No | 29 | 12 |  |  |  |
|  |  |  |  | Survival |  |  |  |  |  |
|  |  |  |  | Yes | 37 | 18 | Chi-square | 0.873 | 0.934 (0.407-2.144) |
|  |  |  |  | No | 33 | 15 |  |  |  |
|  |  |  |  | GVHD3-4 |  |  |  |  |  |
|  |  |  |  | Yes | 13 | 9 | Chi-square | 0.315 | 0.608 (0.230-1.612) |
|  |  |  |  | No | 56 | 24 |  |  |  |
| rs1536215 | TRIM27 | Chr 6: 28900138 | rs209130 | CMV | Matched | Unmatched |  |  |  |
|  | ~3k downstream |  |  | Yes | 47 | 15 | Chi-square | 0.982 | 1.011 (0.403-2.536) |
|  |  |  |  | No | 30 | 10 |  |  |  |
|  |  |  |  | Survival |  |  |  |  |  |
|  |  |  |  | Yes | 39 | 15 | Chi-square | 0.447 | 0.702 (0.281-1.752) |
|  |  |  |  | No | 38 | 10 |  |  |  |
|  |  |  |  | GVHD3-4 |  |  |  |  |  |
|  |  |  |  | Yes | 15 | 7 | Chi-square | 0.352 | 0.612 (0.217-1.730) |
|  |  |  |  | No | 62 | 18 |  |  |  |
| rs139791445 | TRIM27 | Chr 6: 28900314 | rs209130 | CMV | Matched | Unmatched |  |  |  |
|  | ~3k downstream |  |  | Yes | 59 | 3 | Fisher's exact test | 1.000 | 1.035 (0.165-6.485) |
|  |  |  |  | No | 38 | 2 |  |  |  |
|  |  |  |  | Survival |  |  |  |  |  |
|  |  |  |  | Yes | 50 | 4 | Fisher's exact test | 0.367 | 0.266 (0.029-2.466) |
|  |  |  |  | No | 47 | 1 |  |  |  |
|  |  |  |  | GVHD3-4 |  |  |  |  |  |
|  |  |  |  | Yes | 20 | 2 | Fisher's exact test | 0.294 | 0.390 (0.061-2.492) |
|  |  |  |  | No | 77 | 3 |  |  |  |
| rs11244 | HLA-DOB | Chr 6: 32812947 | rs2071479 | CMV | Matched | Unmatched |  |  |  |
|  | 3’UTR |  |  | Yes | 43 | 19 | Chi-square | 0.844 | 1.090 (0.464-2.560) |
|  |  |  |  | No | 27 | 13 |  |  |  |
|  |  |  |  | Survival |  |  |  |  |  |
|  |  |  |  | Yes | 35 | 18 | Chi-square | 0.558 | 0.778 (0.335-1.803) |
|  |  |  |  | No | 35 | 14 |  |  |  |
|  |  |  |  | GVHD3-4 |  |  |  |  |  |
|  |  |  |  | Yes | 13 | 9 | Chi-square | 0.276 | 0.583 (0.219-1.550) |
|  |  |  |  | No | 57 | 22 |  |  |  |
| rs2070120 | HLA-DOB | Chr 6: 32813137 | rs2071479 | CMV | Matched | Unmatched |  |  |  |
|  | 3’UTR |  |  | Yes | 51 | 11 | Chi-square | 0.142 | 0.376 (0.098-1.443) |
|  |  |  |  | No | 37 | 3 |  |  |  |
|  |  |  |  | Survival |  |  |  |  |  |
|  |  |  |  | Yes | 47 | 6 | Chi-square | 0.463 | 1.528 (0.490-4.771) |
|  |  |  |  | No | 41 | 8 |  |  |  |
|  |  |  |  | GVHD3-4 |  |  |  |  |  |
|  |  |  |  | Yes | 20 | 2 | Fisher's exact test | 0.729 | 1.765 (0.364-8.549) |
|  |  |  |  | No | 68 | 12 |  |  |  |
| rs56150445 | HLA-DOB | Chr 6: 32813158-  32813160 | rs2071479 | CMV | Matched | Unmatched |  |  |  |
|  | 3’UTR |  |  | Yes | 62 | . | Fisher's exact test | 0.151 | NA |
|  |  |  |  | No | 38 | 2 |  |  |  |
|  |  |  |  | Survival |  |  |  |  |  |
|  |  |  |  | Yes | 52 | 1 | Fisher's exact test | 1.000 | 1.083 (0.066-17.805) |
|  |  |  |  | No | 48 | 1 |  |  |  |
|  |  |  |  | GVHD3-4 |  |  |  |  |  |
|  |  |  |  | Yes | 22 | . | Fisher's exact test | 1.000 | NA |
|  |  |  |  | No | 78 | 1 |  |  |  |
| rs41258084 | HLA-DOB | Chr 6: 32813180 | rs2071479 | CMV | Matched | Unmatched |  |  |  |
|  | 3’UTR |  |  | Yes | 58 | 4 | Fisher's exact test | 0.179 | 2.636 (0.694-10.022) |
|  |  |  |  | No | 33 | 6 |  |  |  |
|  |  |  |  | Survival |  |  |  |  |  |
|  |  |  |  | Yes | 47 | 4 | Fisher's exact test | 1.000 | 1.068 (0.289-3.943) |
|  |  |  |  | No | 44 | 5 |  |  |  |
|  |  |  |  | GVHD3-4 |  |  |  |  |  |
|  |  |  |  | Yes | 20 | 2 | Fisher's exact test | 1.000 | 1.127 (0.221-5.734) |
|  |  |  |  | No | 71 | 8 |  |  |  |
| rs17220087 | HLA-DOB | Chr 6: 32813299 | rs2071479 | CMV | Matched | Unmatched |  |  |  |
|  | intron |  |  | Yes | 52 | 10 | Fisher's exact test | 0.122 | 0.281 (0.058-1.359) |
|  |  |  |  | No | 37 | 2 |  |  |  |
|  |  |  |  | Survival |  |  |  |  |  |
|  |  |  |  | Yes | 46 | 6 | Chi-square | 0.913 | 1.070 (0.320-3.571) |
|  |  |  |  | No | 43 | 3 |  |  |  |
|  |  |  |  | GVHD3-4 |  |  |  |  |  |
|  |  |  |  | Yes | 20 | 2 | Fisher's exact test | 1.000 | 1.449 (0.293-7.162) |
|  |  |  |  | No | 69 | 10 |  |  |  |
| rs2071479 | HLA-DOB | Chr 6: 32813335 | rs2071479 | CMV | Matched | Unmatched |  |  |  |
|  | intron |  |  | Yes | 59 | 3 | Fisher's exact test | 0.255 | 2.892 (0.650-12.862) |
|  |  |  |  | No | 34 | 5 |  |  |  |
|  |  |  |  | Survival |  |  |  |  |  |
|  |  |  |  | Yes | 47 | 5 | Fisher's exact test | 0.716 | 0.613 (0.138-2.715) |
|  |  |  |  | No | 46 | 3 |  |  |  |
|  |  |  |  | GVHD3-4 |  |  |  |  |  |
|  |  |  |  | Yes | 18 | 4 | Fisher's exact test | 0.066 | 0.240 (0.055-1.053) |
|  |  |  |  | No | 75 | 4 |  |  |  |
| rs17213693 | HLA-DOB | Chr 6: 32813344 | rs2071479 | Survival |  |  |  |  |  |
|  | intron |  |  | Yes | 44 | 8 | Chi-square | 0.648 | 0.767 (0.246-2.397) |
|  |  |  |  | No | 43 | 6 |  |  |  |
|  |  |  |  | GVHD3-4 |  |  |  |  |  |
|  |  |  |  | Yes | 20 | 2 | Fisher's exact test | 0.729 | 1.791 (0.370-8.679) |
|  |  |  |  | NO | 67 | 12 |  |  |  |

Fisher's exact test was used when more than 20% cells had expected count less than 5 for Chi-square test. The unmatched was used as a standard for odds ratio in here. In other words, the chance for getting CMV infection, severe GVHD and survival in the matched genotype in donor-recipient pairs compared to those unmatched.
